# From diagnosis to treatment: patterns in disease-modifying therapy initiation in multiple sclerosis

**DOI:** 10.1177/17562864251398472

**Published:** 2025-12-05

**Authors:** Stefania Iaquinto, Mina Stanikić, Enriqueta Vallejo-Yagüe, Jens Kuhle, Zina-Mary Manjaly, Patrick Roth, Pasquale Calabrese, Chiara Zecca, Milo A. Puhan, Viktor von Wyl

**Affiliations:** Epidemiology, Epidemiology, Biostatistics and Prevention Institute, University of Zurich, Zurich, Switzerland; Department of Epidemiology, Epidemiology, Biostatistics and Prevention Institute, University of Zurich, Zurich, Switzerland; Institute for Implementation Science in Health Care, University of Zurich, Zurich, Switzerland; Epidemiology, Epidemiology, Biostatistics and Prevention Institute, University of Zurich, Zurich, Switzerland; Department of Neurology, University Hospital Basel and University of Basel, Basel, Switzerland; Neurology Clinic and Policlinic, Departments of Head, Spine and Neuromedicine, MS Center and Research Center for Clinical Neuroimmunology and Neuroscience Basel (RC2NB), Clinical Research and Biomedical Engineering, University Hospital Basel and University of Basel, Basel, Switzerland; Department of Neurology, Schulthess Clinic, Zurich, Switzerland; Department of Health Sciences and Technology, ETH Zurich, Zurich, Switzerland; Department of Neurology, University Hospital Zurich, Zurich, Switzerland; Neuropsychology and Behavioral Neurology Unit, Division of Cognitive and Molecular Neuroscience, University of Basel, Basel, Switzerland; Department of Neurology, Neurocenter of Southern Switzerland, Ospedale Regionale di Lugano, EOC, Lugano, Switzerland; Faculty of Biomedical Sciences, Università della Svizzera Italiana, Lugano, Switzerland; Epidemiology, Epidemiology, Biostatistics and Prevention Institute, University of Zurich, Zurich, Switzerland; Department of Epidemiology, Biostatistics and Prevention Institute, University of Zurich, Hirschengraben 84, Zurich 8001, Switzerland Institute for Implementation Science in Health Care, University of Zurich, Zurich, Switzerland

**Keywords:** disease-modifying therapy, multiple sclerosis, patient-reported outcomes, suspected adverse drug reactions

## Abstract

**Background:**

Over recent decades, several high-efficacy disease-modifying therapies (DMTs) have been approved for multiple sclerosis (MS), prompting a shift from an escalation strategy toward early high-efficacy treatment. Yet, DMT initiation patterns and safety profiles remain unexplored in research relying solely on patient self-reports and have not been examined in Switzerland.

**Objectives:**

To investigate (1) time from MS diagnosis to first DMT, (2) temporal trends in initial DMT use, (3) number of suspected adverse drug reactions (sADRs) following the first DMT, and (4) occurrence of any severe sADR.

**Design:**

Retrospective cohort study using longitudinal, patient-reported data from the Swiss MS Registry (SMSR) between 1995 and 2024.

**Methods:**

We analyzed data from SMSR participants with relapsing-remitting MS or clinically isolated syndrome diagnosed after 1995. Time from diagnosis to first DMT was analyzed using Kaplan–Meier analysis. Temporal trends were assessed through annual distributions of first DMTs. sADR burden was modeled with a two-part zero-inflated negative-binomial model, and severe sADRs (⩾1 severe sADR) were assessed with logistic regression. All models were adjusted for sex and age at DMT initiation.

**Results:**

Median time from diagnosis to first DMT decreased from 4.5 months (95% confidence interval (CI): 2–6) in the 1995–2004 diagnosis group to 2 months (95% CI: 1–2) in more recent groups (⩾2010). First DMT use shifted from predominantly low-efficacy to moderate (2013–2020) and, since 2020, to high-efficacy DMTs. Compared to low-efficacy DMTs, moderate (incidence risk ratio (IRR) = 0.63, 95% CI: 0.48–0.84) and high-efficacy DMTs (IRR = 0.55, 95% CI: 0.31–0.98) were associated with fewer sADRs, and high-efficacy DMTs had higher odds of reporting zero sADRs (OR = 8.61, 95% CI: 2.34–31.76). We observed signals of a higher likelihood of severe sADRs in high-efficacy DMTs (OR = 1.41, 95% CI: 0.55–3.24) compared to low-efficacy DMTs, although this was not statistically significant.

**Conclusion:**

Patient self-reports can reliably capture trends in DMT use. In Switzerland, the first DMT use has evolved, with patients starting DMTs earlier and more frequently with higher-efficacy DMTs. While moderate and high-efficacy DMTs were associated with fewer sADRs, they may also carry a higher risk of severe sADRs, underscoring the need to balance personal preferences, efficacy, and safety in first-line MS treatment.

## Introduction

Disease-modifying therapies (DMTs) have demonstrated efficacy in reducing disease activity, delaying disability progression, and improving long-term outcomes in multiple sclerosis (MS). The growing number of available DMTs with varying mechanisms of action, efficacy, and safety profiles^1^ has led to evolving treatment strategies, particularly regarding the timing and selection of the initial DMT.

Traditionally, treatment strategies in relapsing-remitting multiple sclerosis (RRMS) have followed an escalation approach, starting with lower-efficacy DMTs and escalating to more potent treatment options if disease activity persists or in case of breakthrough disease activity. This approach prioritizes patient safety by minimizing exposure to rare but potentially severe side effects associated with high-efficacy DMTs.^2^ In more recent years, however, with the emergence of novel DMTs, there has been a paradigm shift toward the early use of high-efficacy DMTs—a strategy often referred to as the “hit hard and early” strategy. This paradigm shift is largely driven by growing evidence that early initiation of high-efficacy DMTs can result in better long-term outcomes, including fewer relapses, reduced disability progression, and lower risk of irreversible central nervous system damage.^3–5^

Such trends have not yet been investigated, relying exclusively on patient self-reported data, nor have they been studied in Switzerland. Therefore, the present study aimed to examine treatment initiation patterns and associated suspected adverse drug reaction (sADR) profiles among persons with multiple sclerosis (pwMS) in Switzerland, using self-reported data from the Swiss Multiple Sclerosis Registry (SMSR). Specifically, we evaluated temporal changes in the duration between MS diagnosis and the start of the first DMT. In addition, we investigated temporal shifts in the choice of the initial DMT. Finally, we identified specific sADRs and quantified the sADR burden associated with the first DMTs.

## Methods

### Study design, data source, and study population

This observational study used data from the SMSR, a nationwide longitudinal cohort study launched in 2016, that enrolls adults with MS or clinically isolated syndrome (CIS) who live and/or receive care in Switzerland. Prospective participants provide signed informed consent before completing a baseline questionnaire. Subsequently, they are invited to continue providing data through semi-annual follow-up questionnaires. All questionnaires are available in both online and paper formats in Switzerland’s official languages (German, French, and Italian). All questionnaires were developed and pre-tested by the Medico-Scientific Advisory Board (MSAB) working group in collaboration with the MS board, which includes persons affected by MS, to ensure clarity, and content validity prior to implementation. The questionnaires used in this study are provided in Supplemental File 2. Detailed information on the SMSR can be found elsewhere.^6,7^

For this study, sociodemographic, disease-related, and health-related variables were obtained from the baseline questionnaires. DMT-related variables were obtained from the baseline questionnaire and, if missing, supplemented with data from follow-up questionnaires when available.

We included participants with complete MS-type information who were diagnosed with RRMS or CIS from January 1995 onward (first year of DMT availability in Switzerland^8^). For the time-to-first-DMT analysis, we excluded participants with inconsistent data, defined as a DMT start date earlier than the MS diagnosis date, or with missing DMT start dates. The analysis of the type of first DMT was restricted to pwMS who had initiated a DMT during the study period. Analyses of sADRs were further limited to those with available self-reported sADR data that could be linked to their corresponding first DMT (i.e., reported at or near the time of DMT initiation and referring specifically to the first DMT). All data collected up to and including November 30, 2024, were included.

This study followed the STROBE (Strengthening the Reporting of Observational Studies in Epidemiology) guidelines for reporting observational research.^9^

### Outcome measures

The outcomes of interest in this study were (1) the time from MS diagnosis to initiation of the first DMT (defined as the number of months between MS diagnosis and initiation of the first DMT); (2) the first DMT (recorded as the pharmaceutical agent and categorized by treatment efficacy as low, moderately, or highly effective); (3) the total count of sADRs; and (4) the occurrence of any severe sADR, both stratified by DMT efficacy category.

MS diagnosis date and first DMT initiation date were obtained from participant self-reports. Both were recorded in month/year format, and the time-to-treatment initiation was calculated as the number of months between these two dates.

All DMT-specific analyses were conducted both at the level of individual DMTs (by active ingredient) and by treatment effectiveness category, with DMTs classified into three categories based on their efficacy: low, moderate, and high. This classification follows the Association of British Neurologists (ABN) guidelines^10^ and primarily reflects efficacy in reducing annualized relapse rate as reported in pivotal trials; it does not capture other important outcomes of relevance to pwMS, such as quality of life. Low-efficacy DMTs included: (peg-)interferon beta-1a/1b, and glatiramer acetate. Moderate-efficacy DMTs included: fingolimod, dimethyl fumarate, diroximel fumarate, ozanimod, daclizumab, ponesimod, siponimod, teriflunomide, and cladribine. High-efficacy DMTs included: ocrelizumab, natalizumab, alemtuzumab, ofatumumab, and rituximab.

sADRs were reported by participants by selecting from a predefined list of ADRs, with the option to describe additional sADRs in a free-text field. Free-text responses were reviewed and recoded into existing categories where applicable or grouped into new categories when necessary. Additionally, for subsequent modeling, sADRs were classified as severe or non-severe. Severe sADRs—defined as opportunistic infections (e.g., herpes infections, chronic pneumonia, oral candidiasis), progressive multifocal leukoencephalopathy (PML), hepatotoxicity, and depression—were identified based both on neurologist expertise and on the adverse-event categorization used in pivotal DMT clinical trials. Non-severe sADRs included tiredness/fatigue, pain, flu-like symptoms, sleep problems, gastrointestinal problems, dizziness, hot flushes, headache, allergic reactions, hair loss, neuropsychiatric symptoms (e.g., mood changes), and skin problems. Only sADRs reported in combination with the first DMT were considered.

### Statistical analysis

Sociodemographic, MS-related, and health-related characteristics were descriptively summarized for each of the distinct study population samples (outcome dependent). Categorical data were summarized using counts and percentages, and continuous variables using medians and interquartile ranges (IQR).

The time from MS diagnosis to initiation of the first DMT was analyzed using Kaplan–Meier survival analysis, with follow-up starting at the date of MS diagnosis and ending at the date of DMT initiation or censored at participants’ last follow-up if no DMT was initiated.

To explore temporal trends in time to DMT initiation, the analysis was stratified by MS diagnosis period, defined according to intervals between revisions of the McDonald diagnostic criteria for MS (up to 2005, 2006–2010, 2011–2016, and 2017 onward). Failure curves were plotted for each diagnosis period to visualize cumulative DMT initiation over time. For additional contextualization of the potential temporal changes in treatment initiation patterns, sociodemographic and health-related characteristics at the time of DMT initiation were descriptively summarized across diagnosis periods. As a sensitivity analysis, we also examined the time from first MS symptoms to DMT initiation (restricted to participants with available data on symptom onset).

To examine temporal trends in the choice of first DMTs after an MS diagnosis, the study period (starting in 1995) was segmented into calendar years, and each participant’s first DMT initiation was assigned to the year it occurred. For each year, we calculated the distribution of all first DMTs, expressed as the proportion of initiations with each specific DMT among all first DMT initiations in that corresponding year. This was done both at the level of individual DMTs and grouped by DMT efficacy (low, moderately, or highly effective).

**Figure 1.**
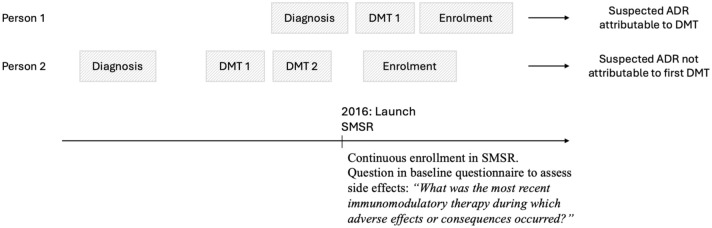
Example timelines illustrating side effect reporting limitations. For individuals who received multiple DMTs before SMSR enrolment, sADRs cannot be attributed to the first DMT, as the baseline questionnaire only captures sADRs related to the most recent DMT. A longer time lag between diagnosis and registry enrollment increases the likelihood of multiple treatments having occurred before baseline, making attribution to the first DMT impossible. ADR, adverse drug reaction; DMT, disease-modifying therapy; sADR, suspected adverse drug reaction; SMSR, Swiss MS Registry.

To assess the burden of sADRs following first DMT initiation, we included all sADRs reported in the first available questionnaire completed after treatment start, which was the baseline questionnaire for the majority of participants. We fitted both a two-part zero-inflated negative-binomial (ZINB) model and a logistic regression model. The ZINB model consisted of a log-link negative-binomial component modeling the total count of sADRs and a logit-link zero-inflation component accounting for excess zeros. Separately, we fitted a logistic regression predicting the odds of experiencing at least one severe sADR (vs no severe sADR). In both models, the DMT efficacy category (low, moderate, high) was the main explanatory variable. The models were additionally adjusted for sex (female or male) and age at treatment initiation. We report incidence risk ratios (IRRs) with 95% confidence intervals (CIs) for the ZINB count component and odds ratios (ORs) with 95% CIs for the ZINB zero-inflated component and for the logistic model. To account for potential selection bias due to differential inclusion (i.e., only participants who had initiated a DMT and had sADR information for their first DMT, reported at or close to DMT initiation, were included in the analysis), we applied inverse probability weighting (IPW). Figure 1 shows the limitation in attributing sADRs to the first DMT, especially among participants with a substantial time gap between MS diagnosis and SMSR enrollment. Propensity scores were obtained from a logistic regression model including sex (female or male), age at treatment initiation, diagnosis period (1995–2004, 2005–2009, 2010–2016, ⩾2017), MS type (CIS or RRMS), and DMT efficacy category (low, moderate, or high efficacy). These covariates were selected based on a comparison of individuals included versus excluded from the sADR analysis (Supplemental Table 1). The resulting scores were used to calculate stabilized weights, which were then applied in both the ZINB and logistic regression models to adjust for differential inclusion. Covariate balance before and after weighting was assessed using standardized mean differences.

All analyses were carried out using R (version 4.4.1, R Foundation for Statistical Computing, Vienna, Austria) and R Studio (version 2024.09.0+375, Posit PBC, Boston, MA, USA). The following additional packages besides base packages were used: dplyr (version 1.1.4), tableone (version 0.13.2), survival (version 3.7.0), survminer (version 0.4.9), ggplot2 (version 3.5.1), and pscl (version 1.5.9).

## Results

**Figure 2.**
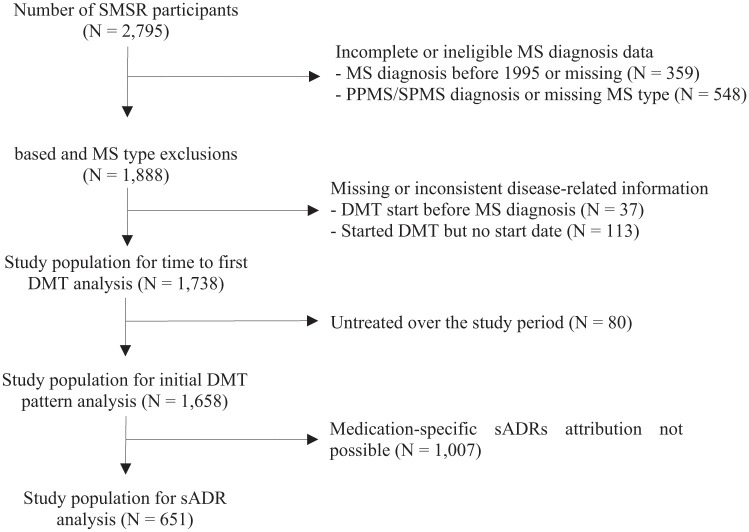
Participants flowchart. DMT, disease-modifying therapy; MS, multiple sclerosis; PPMS, primary progressive MS; sADR, suspected adverse drug reaction; SMSR, Swiss Multiple Sclerosis Registry; SPMS, secondary progressive MS.

Among the 2795 participants enrolled in the SMSR by the end of November 2024, 1888 met the inclusion criteria based on diagnosis year and MS type (Figure 2). After excluding individuals with missing or inconsistent treatment-related information, 1738 were included in the analysis of time to first DMT. For the analysis of the first DMT choice, 1658 participants who started a DMT throughout the study period and who had complete treatment information were included. Furthermore, 651 individuals, for whom information on sADRs was available, were included in the sADR analysis.

Table 1 summarizes the demographic and health-related characteristics of the SMSR study populations used to address the different research aims. Across all samples, the majority were female (75.6%–77.0%), with a median age at MS diagnosis of 35.0 years (IQR 28.0–43.0) in the time-to-treatment and DMT-type subsamples (IQR 28.0–42.0) and 36.0 years (IQR 30.0–44.0) in the sADR subsample. Median age at diagnosis and at first DMT was comparable between groups (ranging from 35 to 36 years), and most participants were diagnosed with RRMS (93.7%–94.5%). The distribution of first DMT efficacy categories differed, with a higher proportion of pwMS in the sADR analysis being treated with a moderate or high-efficacy DMT as their first DMT (53.9% and 21.2%), which coincides with a higher proportion of individuals in the sADR analysis diagnosed in 2017 or later (54.8%), compared to the other samples (28.5% and 28.8%).

**Table 1.** Characteristics of pwMS used in the three analytic samples.

Characteristics	Time-to-treatment analysis	DMT-type analysis	Suspected ADR analysis
*N* (%)	1738 (100)	1658 (100)	651 (100)
Female sex, *N* (%)	1333 (76.7)	1277 (77.0)	492 (75.6)
Swiss citizenship, *N* (%)	1515 (87.2)	1445 (87.2)	563 (86.5)
Highest education, *N* (%)			
Mandatory, high school, apprenticeship	750 (43.2)	723 (43.6)	261 (40.1)
Higher professional education	198 (11.4)	196 (11.8)	95 (14.6)
University, applied university	417 (24.0)	398 (24.0)	190 (29.2)
Missing	373 (21.5)	341 (20.6)	105 (16.1)
Diagnosis period, *N* (%)			
1995–2004	314 (18.1)	288 (17.4)	30 (4.6)
2005–2009	290 (16.7)	284 (17.1)	37 (5.7)
2010–2016	634 (36.5)	613 (37.0)	227 (34.9)
⩾2017	500 (28.8)	473 (28.5)	357 (54.8)
Age at first symptoms, median (IQR)	31.00 (25.00, 39.00)	31.00 (25.00, 39.00)	33.00 (27.00, 41.00)
Missing	74 (4.3)	69 (4.2)	33 (5.1)
Age at diagnosis, median (IQR)	35.00 (28.00, 43.00)	35.00 (28.00, 42.00)	36.00 (29.00, 43.00)
Age at first DMT, median (IQR)	35.00 (28.00, 43.00)	35.00 (28.00, 43.00)	36.00 (30.00, 44.00)
Not started DMT in observation period	80 (4.6)	—	—
MS type, *N* (%)			
Clinically isolated syndrome	95 (5.5)	78 (4.7)	41 (6.3)
Relapsing-remitting MS	1643 (94.5)	1580 (95.3)	610 (93.7)
First DMT category, *N* (%)			
High	186 (10.7)	186 (11.2)	138 (21.2)
Moderate	541 (31.1)	541 (32.6)	351 (53.9)
Low	931 (53.6)	931 (56.2)	162 (24.9)
Untreated in observation period	80 (4.6)	-	-
Comorbidities, *N* (%)			
No	710 (40.9)	678 (40.9)	301 (46.2)
Yes (⩾1)	658 (37.9)	641 (38.7)	246 (37.8)
Missing	370 (21.3)	339 (20.5)	104 (16.0)
Diagnosis setting, *N* (%)			
General practitioner	20 (1.2)	19 (1.1)	6 (0.9)
Neurological practice	659 (37.9)	630 (38.0)	228 (35.0)
Hospital	844 (48.6)	801 (48.3)	315 (48.4)
Other	12 (0.7)	11 (0.7)	4 (0.6)
Missing	203 (11.7)	197 (11.9)	98 (15.1)

Column 1 shows pwMS included in the time-to-first DMT analysis; column 2 shows those included in the analysis of DMT initiation patterns; and column 3 shows participants included in the side effects analysis.

ADR, adverse drug reaction; DMT, disease-modifying therapy; MS, multiple sclerosis; pwMS, persons with multiple sclerosis.

### Time to first DMT

Overall, 80 pwMS (4.6%) never started a DMT during follow-up. Among the 1658 pwMS who started a DMT, 139 (8.4%) initiated their first DMT ⩾24 months after diagnosis, with a decrease in proportion from 24.7% in the 1995–2004 group to just 1.1% in the ⩾2017 group. Furthermore, 12.8% of participants started DMT more than one year after diagnosis, with proportions decreasing from 32.3% in the 1995–2004 group to 3.2% in the ⩾2017 group.

While participants characteristics across the four diagnosis period groups were largely comparable, there was a trend toward older age at symptom onset and diagnosis in more recent groups: median age at symptom onset rose from 28.0 years (IQR 24.0–35.0) in the 1995–2004 group to 33.0 years (IQR 27.0–40.0) in the ⩾2017 group, and median age at diagnosis increased from 32.0 years (IQR 26.0–38.0) to 35.0 years (IQR 29.0–43.0) over the same periods (Supplemental Table 2).

**Figure 3.**
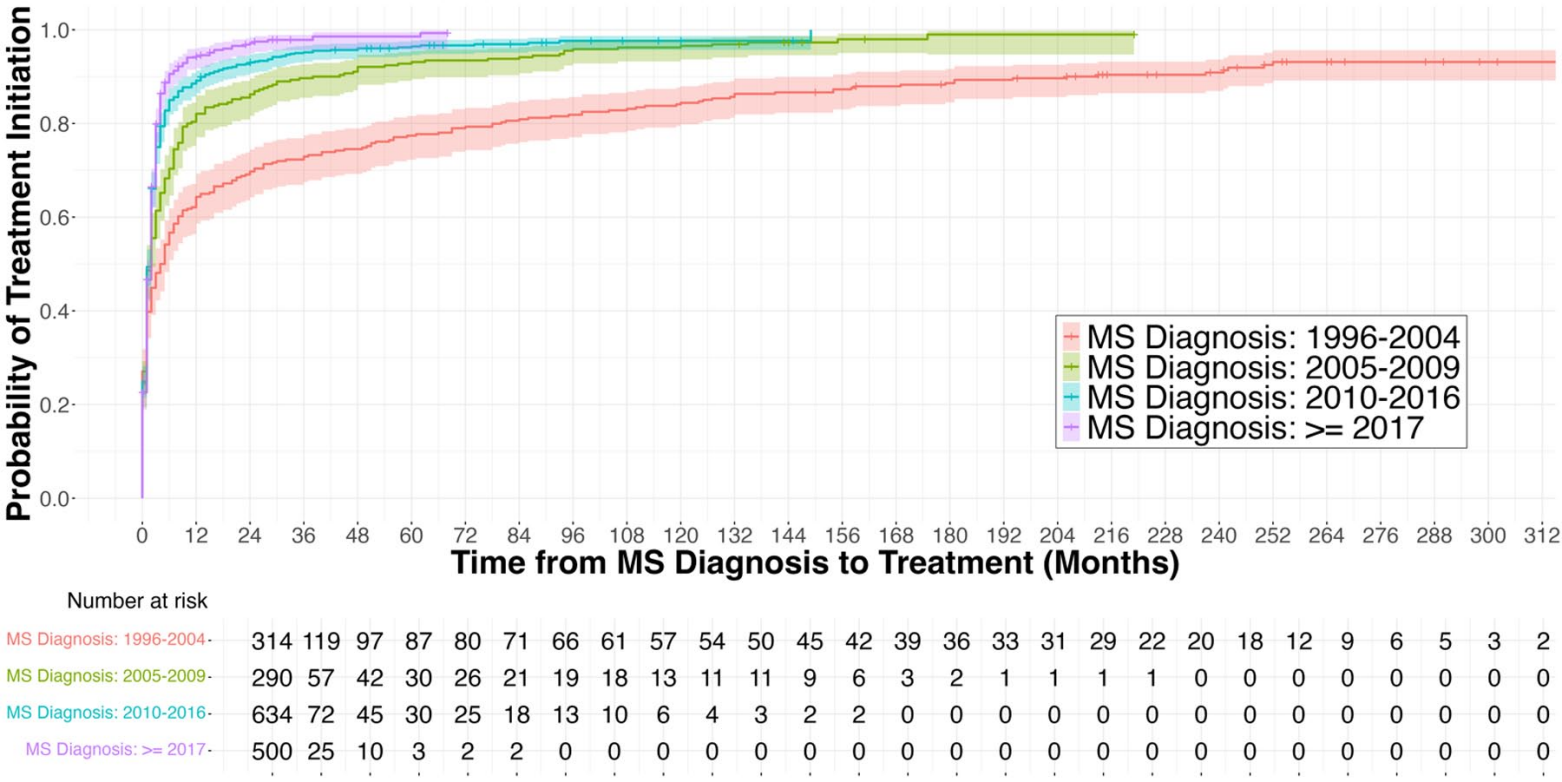
Time from MS diagnosis to first DMT by diagnosis period. The failure curves, derived from Kaplan–Meier analysis, display the cumulative probability of treatment initiation over time since MS diagnosis, stratified by diagnosis period: 1995–2004, 2005–2009, 2010–2016, and ⩾2017. Shaded areas represent 95% confidence intervals. DMT, disease-modifying therapy; MS, multiple sclerosis.

Kaplan–Meier survival analysis showed a decrease in time from MS diagnosis to first DMT over successive diagnosis periods (Figure 3). Patients diagnosed in more recent periods (⩾2017 and 2010–2016) initiated treatment faster compared to those diagnosed in earlier periods (2005–2009 and 1995–2004). The median time to first DMT decreased from 4.5 months (95% CI: 2–6) in the 1995–2004 diagnosis group to 2 months (95% CI: 1–2) in both the 2010–2016 and ⩾2017 diagnosis groups. The same pattern was observed in the sensitivity analysis using time from first MS symptoms onset to DMT initiation (based on *N* = 1567 pwMS; Supplemental Figure 1).

### Type of first DMT

**Figure 4.**
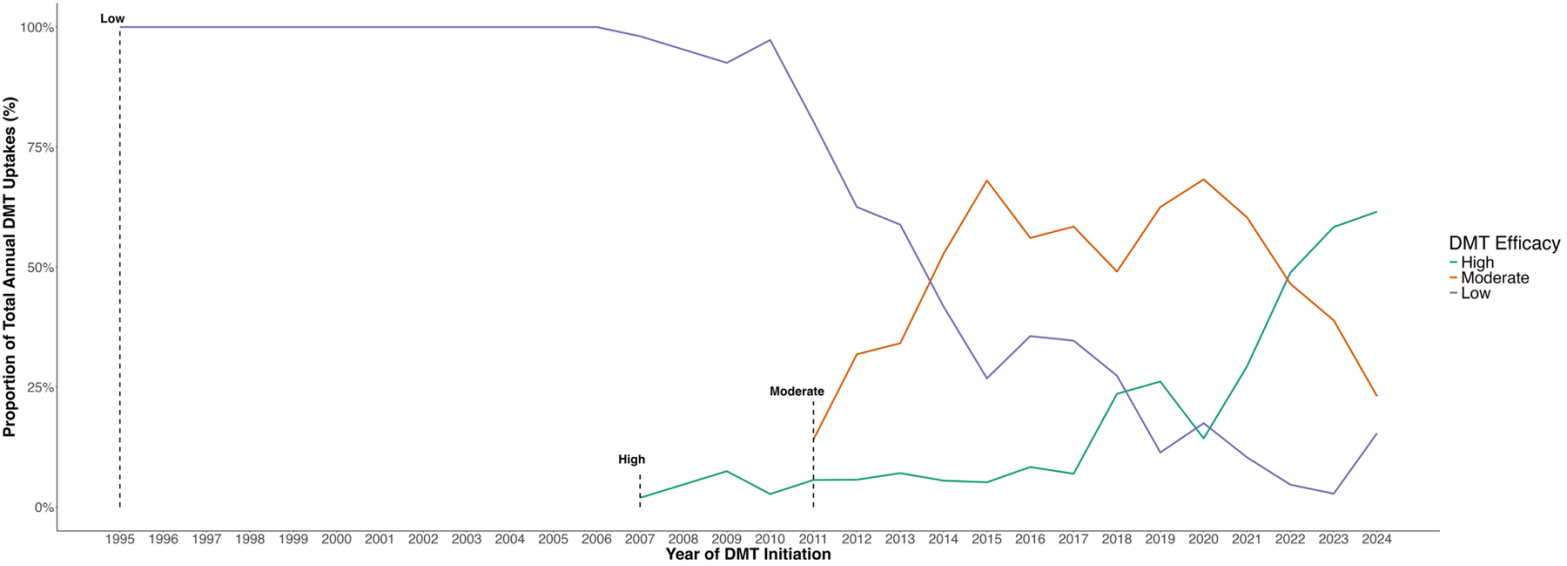
Temporal trends in the uptake of DMTs for MS by efficacy category from 1995 to 2024. Each colored line represents the annual proportion of total DMT initiations, stratified by efficacy category: low (blue), moderate (orange), and high (green). Vertical dashed lines indicate the year when the first DMT from the corresponding efficacy category was introduced in Switzerland. DMT, disease-modifying therapy; MS, multiple sclerosis.

Temporal trends in the choice of the first DMT categories are presented in Figure 4, with Supplemental Figure 2 detailing the use of individual DMTs. The corresponding numerical data can be found in Supplemental Table 3. The use of low-efficacy DMTs as first-line treatment began to decline from 2010 onwards, following a decade of exclusive use. The decline coincided with the increasing uptake of moderate-efficacy DMTs, which became the predominant choice between 2013 and 2020. From 2020, initiations of high-efficacy DMTs rose sharply, overtaking moderate-efficacy DMTs by 2022. By 2024, high-efficacy DMTs represented the majority of first DMT initiations, while low-efficacy DMTs were used the least. Similarly, when looking at different diagnosis period groups, initiation of moderate-efficacy DMTs increased from 12.1% in the 1995–2004 group to 57.8% in the ⩾2017 group, and high-efficacy use increased from 1.9% to 25.8%, while the proportion of low-efficacy DMTs declined from 86.0% to 16.4% (Supplemental Table 2).

### Suspected ADRs

Table 2 shows descriptive frequencies of reported sADRs by DMT efficacy group, while Supplemental Table 4 presents a more detailed breakdown of sADRs by pharmacological DMT class. As shown in Table 2, flu-like symptoms, headache, and fatigue were most frequently reported in the low-efficacy group, whereas gastrointestinal problems, hot flushes, and hair loss were more commonly reported among users of moderate-efficacy DMTs. Supplemental Table 4 further shows that flu-like symptoms and headache were primarily reported with interferons, gastrointestinal problems and hot flushes with fumarates, and hair loss with teriflunomide. Opportunistic infections were most frequently reported with cladribine and anti-CD20 therapies. The proportion of pwMS reporting at least one severe sADRs was similar across groups, with a slightly higher proportion in the high-efficacy DMT group (9.4%) compared to the moderate- and low-efficacy DMT group (7.1% and 6.8%).

**Table 2.** Frequency of reported sADR by DMT efficacy category.

Characteristics	DMT efficacy			
	Overall	Low	Moderate	High
*N*	651	162	351	138
sADRs, *N* (%)				
Non-severe sADR				
Pain	2 (0.3)	2 (1.2)	0 (0.0)	0 (0.0)
Flu-like symptoms	132 (20.3)	78 (48.1)	34 (9.7)	20 (14.5)
Headache	111 (17.1)	58 (35.8)	36 (10.3)	17 (12.3)
Gastrointestinal problems	48 (7.4)	10 (6.2)	36 (10.3)	3 (2.1)
Sleep problems	71 (10.9)	30 (18.5)	30 (8.5)	11 (8.0)
Fatigue	135 (20.7)	50 (30.9)	64 (18.2)	22 (15.9)
Skin problems	3 (0.5)	2 (1.2)	1 (0.3)	0 (0.0)
Allergic reactions	11 (1.7)	3 (1.9)	4 (1.1)	4 (2.9)
Hot flushes	139 (21.4)	36 (22.2)	87 (24.8)	16 (11.6)
Hair loss	61 (9.4)	17 (10.5)	38 (10.8)	6 (4.3)
Dizziness	12 (1.8)	3 (1.9)	6 (1.7)	3 (2.1)
Neuropsychiatric symptoms	48 (7.4)	15 (9.3)	27 (7.7)	6 (4.3)
Severe sADR				
Hepatotoxicity	1 (0.2)	0 (0.0)	1 (0.3)	0 (0.0)
PML	4 (0.6)	0 (0.0)	2 (0.6)	2 (1.4)
Opportunistic infections	21 (3.2)	1 (0.6)	11 (3.1)	9 (5.8)
Depression	33 (5.1)	11 (6.8)	17 (4.8)	5 (3.6)
Reported any severe sADR, *N* (%)	49 (7.5)	11 (6.8)	25 (7.1)	13 (9.4)
Number of sADRs, mean (SD)	1.30 (2.05)	1.98 (2.22)	1.13 (1.95)	0.92 (1.92)

ADR, adverse drug reaction; DMT, disease-modifying therapy; PML, progressive multifocal leukoencephalopathy; sADR, suspected adverse drug reaction; SD, standard deviation.

**Figure 5.**
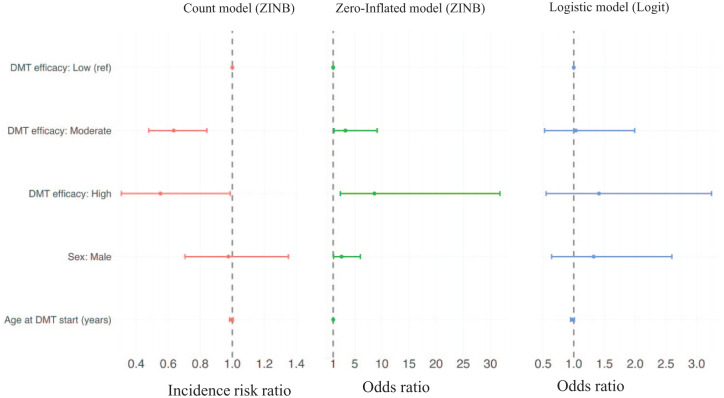
Estimated effects (point estimates and 95% CIs) on sADR burden and severity, based on IPW models. The left panel shows the IRRs from the negative-binomial component of the ZINB model predicting the total number of sADRs; the middle panel shows the ORs from the logistic zero-inflation component of the ZINB model estimating the probability of reporting no sADRs; and the right panel shows the ORs from a separate logistic regression model predicting the odds of experiencing at least one severe sADR. sADR, suspected adverse drug reaction; CI, confidence interval; DMT, disease-modifying therapy; IPW, inverse probability weighted; IRR, incidence risk ratio; OR, odds ratio; ZINB, zero-inflated negative binomial.

In the ZINB model for the total sADR count (left panel, Figure 5), both moderate-efficacy (IRR = 0.63, 95% CI: 0.48–0.84) and high-efficacy (IRR = 0.55, 95% CI: 0.31–0.98) DMTs were associated with fewer sADRs compared to low-efficacy DMTs. In the zero-inflation component of the ZINB model (middle panel, Figure 5), high-efficacy DMTs were associated with 8.6 times higher odds of reporting zero sADRs compared with low-efficacy DMTs (OR = 8.61; 95% CI 2.34–31.76), and moderate-efficacy DMTs showed 3.3 times higher odds (OR = 3.26; 95% CI 1.16–9.12). In the logistic regression model of sADR severity (right panel, Figure 5), moderate-efficacy DMTs were associated with 3% higher odds (OR = 1.03, 95% CI 0.52–1.99) and high-efficacy DMTs with 41% higher odds (OR = 1.41, 95% CI 0.55–3.24) of experiencing at least one severe sADR compared with low-efficacy DMTs; however, neither estimate was statistically significant. The results of the IPW models were consistent in direction and similar in magnitude to those of the unweighted models.

## Discussion

This study examined trends in DMT initiation patterns among pwMS in Switzerland using nationwide data from the SMSR. Our results indicate a decrease in the time between MS diagnosis and initiation of the first DMT from the earliest to the most recent diagnosis groups. Concurrently, prescribing patterns shifted from predominantly low-efficacy DMTs toward moderate-efficacy DMTs, and more recently, toward high-efficacy DMTs. Moderate- and high-efficacy DMTs were associated with fewer sADRs and a higher probability of reporting no sADRs compared to low-efficacy DMTs. While the severity of sADRs was not associated with DMT efficacy, high-efficacy DMTs showed a potential signal of slightly elevated odds compared to low-efficacy DMTs.

Our study shows that in Switzerland, pwMS diagnosed in more recent years not only start treatment earlier but are also more often on high-efficacy DMTs from the outset. This approach is increasingly supported by evidence showing that early use of high-efficacy DMTs can reduce relapse rates, delay disability accumulation, and improve long-term outcomes, along with growing evidence supporting their safety profile.^3–5,11^

The observed decrease in time from MS diagnosis to first DMT initiation, as also reported by several other studies,^12,13^ likely reflects a shift in clinical practice and MS management toward earlier therapeutic intervention.^2,14,15^ Increased awareness among clinicians and pwMS about the benefits of early treatment, combined with greater accessibility, potentially better-tolerated therapies,^1^ and an expanding array of available DMTs, has likely contributed to the observed trend toward faster treatment initiation after diagnosis. Our sensitivity analysis further supports this, showing a similar decrease in time from first symptom onset to DMT initiation across diagnosis period groups—likely a result of successive revisions of the diagnostic criteria, which have facilitated earlier diagnosis of MS and, consequently, more timely DMT initiation.^16^ Beyond earlier treatment initiation, our findings also point toward a shift in choice of first-line DMTs, aligning with observations from other studies of DMT prescriptions in the US, Germany, and France.^17–19^ We noticed a transition from low-efficacy DMTs toward increasing use of moderate- and high-efficacy DMTs as initial treatments after an MS diagnosis. While this shift may partly be explained by the increasing availability of new treatment options, the continued increase in high-efficacy DMT use even after a broad range of options became available suggests a growing preference for a “hit hard and early” strategy that emphasizes early, intensive disease management to prevent irreversible neurological damage.^14,20^

In addition to clinical and diagnostic developments, several contextual factors have likely contributed to this shift. The Swiss healthcare system offers relatively low barriers to accessing DMTs, with all approved therapies reimbursed by mandatory health insurance, and neurologists having broad prescribing authority, enabling rapid and widespread adoption of new therapies once approved. Furthermore, national guidelines for immunotherapy (published in 2022) developed by a joint expert group mandated by the Swiss MS Society and the Swiss Neurological Society, recommend early DMT initiation following diagnosis and emphasize a personalized treatment strategy balancing efficacy and safety considerations,^21^ which is also in line with the European recommendations from the European Academy of Neurology (EAN) and the European Committee for Treatment and Research in Multiple Sclerosis (ECTRIMS).^22^

Treatment decisions should not only prioritize early, high-efficacy initiation of DMTs but also carefully balance efficacy with safety.^20^ Our findings on sADRs following first DMT initiation showed that moderate- and high-efficacy DMTs were associated with fewer sADRs compared to low-efficacy DMTs. At the same time, we observe a statistically non-significant signal toward a higher likelihood of severe sADRs among users of high-efficacy DMTs. This is consistent with existing literature showing that newer high-efficacy DMTs, while potentially carrying a smaller overall side effect burden in comparison to low-efficacy DMTs, pose a higher risk for rare but serious adverse events (e.g., severe infections and PML).^23,24^ Low-efficacy DMTs, by contrast, may cause more frequent, milder, yet daily burdensome side effects (e.g., flu-like symptoms and hot flushes). Taken together, these findings underscore the importance of individualized and careful risk-benefit assessment in MS treatment.

Finally, our results replicate DMT initiation patterns and safety profiles that have previously been reported in studies relying on both clinician-entered and patient-reported data. In contrast, our study derived these findings using exclusively self-reported information, demonstrating that patient-reported registries can capture detailed and clinically relevant treatment patterns and safety outcomes. This underscores the broader applicability of self-reported registry data for MS research worldwide. Beyond the immediate context of DMT use, our findings also highlight the potential of self-reported registry data to contribute to pharmacovigilance (PV). In Switzerland, patients and caregivers can directly report sADRs to the national regulatory authority, Swissmedic, although this probably remains less common than reporting by healthcare professionals. Moreover, spontaneous ADR reporting in Switzerland is generally underreported, which limits the completeness of the data. Our study shows that patient-reported data can complement traditional PV systems, add new information and perspectives about sADRs, and strengthen the role of patients in monitoring drug safety.

This study has some limitations that should be considered when interpreting the findings. The analysis of sADRs may be influenced by confounding by indication, whereby treatment choice may vary according to product-specific recommendations, contraindications, or known side effect profiles, and thus be linked to patient characteristics. Furthermore, while the analysis of sADRs may be subject to selection bias from varying inclusion, we incorporated IPWs to attenuate its impact. PwMS who experienced severe sADRs, such as opportunistic infections, may be underrepresented in the SMSR; however, the opposite could also be true, with such experiences motivating participation. Consequently, our results may under- or overestimate the frequency and impact of severe sADRs associated with certain DMTs. However, the SMSR is designed as a flexible, long-term observational registry,^6,7^ allowing participants to pause and later resume their involvement. The design accommodates fluctuations in health status and enables individuals to skip one or more follow-ups and still contribute data at a later point, for example, regarding sADRs they experienced in the past. Moreover, while participants explicitly attributed their reported sADRs to specific DMTs, some sADRs, such as fatigue or pain, are also common manifestations of MS itself. As such, it may not always be possible to clearly distinguish between true medication-related sADRs and symptoms stemming from the disease. Finally, due to the absence of detailed information on the intensity of individual sADRs, the classification into severe and non-severe categories may be imprecise, with the potential for both under- and overestimation of actual severity. Moreover, by using a mere quantitative approach to enumerate sADRs, the clinical significance of severe adverse events might be overshadowed.

In conclusion, our findings indicate a trend toward earlier and more aggressive DMT initiation among pwMS in the SMSR, likely driven by increased drug availability, drug-related evidence, refined clinical treatment regimes, improved diagnostic capabilities, and favorable healthcare infrastructure. These patterns were identified using self-reported data only, underscoring the ability of patient-reported registries to capture detailed and clinically relevant treatment patterns and safety outcomes. As the treatment landscape moves toward earlier use of high-efficacy DMTs, shared decision-making becomes increasingly important, ensuring individualized treatment approaches that carefully balance efficacy, safety, and patient preferences.

## Supplemental Material

sj-docx-1-tan-10.1177_17562864251398472 – Supplemental material for From diagnosis to treatment: patterns in disease-modifying therapy initiation in multiple sclerosis Supplemental material, sj-docx-1-tan-10.1177_17562864251398472 for From diagnosis to treatment: patterns in disease-modifying therapy initiation in multiple sclerosis by Stefania Iaquinto, Mina Stanikić, Enriqueta Vallejo-Yagüe, Jens Kuhle, Zina-Mary Manjaly, Patrick Roth, Pasquale Calabrese, Chiara Zecca, Milo A. Puhan and Viktor von Wyl in Therapeutic Advances in Neurological Disorders

sj-docx-2-tan-10.1177_17562864251398472 – Supplemental material for From diagnosis to treatment: patterns in disease-modifying therapy initiation in multiple sclerosis Supplemental material, sj-docx-2-tan-10.1177_17562864251398472 for From diagnosis to treatment: patterns in disease-modifying therapy initiation in multiple sclerosis by Stefania Iaquinto, Mina Stanikić, Enriqueta Vallejo-Yagüe, Jens Kuhle, Zina-Mary Manjaly, Patrick Roth, Pasquale Calabrese, Chiara Zecca, Milo A. Puhan and Viktor von Wyl in Therapeutic Advances in Neurological Disorders

sj-docx-3-tan-10.1177_17562864251398472 – Supplemental material for From diagnosis to treatment: patterns in disease-modifying therapy initiation in multiple sclerosis Supplemental material, sj-docx-3-tan-10.1177_17562864251398472 for From diagnosis to treatment: patterns in disease-modifying therapy initiation in multiple sclerosis by Stefania Iaquinto, Mina Stanikić, Enriqueta Vallejo-Yagüe, Jens Kuhle, Zina-Mary Manjaly, Patrick Roth, Pasquale Calabrese, Chiara Zecca, Milo A. Puhan and Viktor von Wyl in Therapeutic Advances in Neurological Disorders

## Declarations

**Ethics approval and consent to participate:** The Swiss Multiple Sclerosis Registry (SMSR) was approved by the Ethics Committee of the Canton of Zurich (PB-2016-00,894; BASEC-NR 2019-01,027). All participants of the SMSR provide written informed consent.

**Consent for publication:** Not applicable.

**Availability of data and materials:** The data that support the findings of this study are not publicly available due to privacy or ethical restrictions, but can be made available upon reasonable request. Requests should be directed to the corresponding author.
