# Supplementary material for: From diagnosis to treatment: patterns in disease-modifying therapy initiation in multiple sclerosis
Source: Ther Adv Neurol Disord. 2025 Dec 5;18:17562864251398472. doi: 10.1177/17562864251398472 (PMC12681631; doi:10.1177/17562864251398472)
Supplement: sj-docx-1-tan-10.1177_17562864251398472 – Supplemental material for From diagnosis to treatment: patterns in disease-modifying therapy initiation in multiple sclerosis [file sj-docx-1-tan-10.1177_17562864251398472.docx]

|  | **PwMS under DMT who were included in the suspected ADR analysis** | **PwMS under DMT who were excluded from the suspected ADR analysis** |
| --- | --- | --- |
| N (%) | 651 (100) | 1007 (100) |
| Female sex, N (%) | 492 (75.6) | 785 (78.0) |
| Swiss citizenship, N (%) | 563 (86.5) | 882 (87.6) |
| Highest education, N (%) |  |  |
| Mandatory, high school, apprenticeship | 261 (40.1) | 462 (45.9) |
| Higher professional education | 95 (14.6) | 101 (10.0) |
| University, applied university | 190 (29.2) | 208 (20.7) |
| Missing | 105 (16.1) | 236 (23.4) |
| Diagnosis period, N (%) |  |  |
| 1995-2004 | 30 (4.6) | 258 (25.6) |
| 2005-2009 | 37 (5.7) | 247 (24.5) |
| 2010-2016 | 227 (34.9) | 386 (38.3) |
| ≥2017 | 357 (54.8) | 116 (11.5) |
| Age at first symptoms, median [IQR] | 33.00 [27.00, 41.00] | 30.00 [25.00, 38.00] |
| Missing | 33 (5.1) | 39 (3.9) |
| Age at diagnosis, median [IQR] | 36.00 [29.00, 43.00] | 34.00 [27.00, 42.00] |
| Age at first DMT, median [IQR] | 36.00 [30.00, 44.00] | 35.00 [27.00, 43.00] |
| MS type, N (%) |  |  |
| Clinically isolated syndrome | 41 (6.3) | 37 (3.7) |
| Relapsing remitting MS | 610 (93.7) | 970 (96.3) |
| First DMT Category, N (%) |  |  |
| High | 138 (21.2) | 48 (4.8) |
| Moderate | 351 (53.9) | 190 (18.9) |
| Low | 162 (24.9) | 769 (76.4) |
| Untreated in observation period | - | - |
| Comorbidities, N (%) |  |  |
| No | 301 (46.2) | 377 (37.4) |
| Yes (≥1) | 246 (37.8) | 395 (39.2) |
| Missing | 104 (16.0) | 235 (23.3) |
| Diagnosis setting, N (%) |  |  |
| General practitioner | 6 (0.9) | 13 (1.3) |
| Neurological practice | 228 (35.0) | 402 (39.9) |
| Hospital | 315 (48.4) | 486 (48.3) |
| Other | 4 (0.6) | 7 (0.7) |
| Missing | 98 (15.1) | 99 (9.8) |

**Supplementary Materials**

**Supplementary Table 1**: Comparison of persons with MS (pwMS) under disease-modifying therapy (DMT) who were included vs. excluded from the suspected adverse drug reaction (ADR) analysis.

|  | **Diagnosis Period** | | | | |
| --- | --- | --- | --- | --- | --- |
|  | **Overall** | **1995-2004** | **2005-2009** | **2010-2016** | **≥2017** |
| N (%) | 1738 (100) | 314 (18.1) | 290 (16.7) | 634 (36.5) | 500 (28.8) |
| Female sex, N (%) | 1333 (76.7) | 440 (76.8) | 237 (81.7) | 486 (76.7) | 369 (73.8) |
| Swiss citizenship, N (%) | 1515 (87.2) | 278 (88.3) | 259 (89.3) | 566 (89.3) | 413 (82.6) |
| Highest education, N (%) |  |  |  |  |  |
| Mandatory, high school,  apprenticeship | 750 (43.2) | 155 (49.2) | 140 (48.3) | 263 (41.5) | 193 (38.6) |
| Higher professional   education | 198 (11.4) | 21 (6.7) | 27 (9.3) | 87 (13.7) | 63 (12.6) |
| University, applied   university | 417 (24.0) | 72 (22.9) | 62 (21.4) | 147 (23.2) | 136 (27.2) |
| Missing | 373 (21.5) | 67 (21.3) | 61 (21.0) | 137 (21.6) | 108 (21.6) |
| Age at first symptoms, median [IQR] | 31.00 [25.00, 39.00] | 28.00 [24.00, 35.00] | 30.00 [24.00, 39.00] | 32.00 [26.00, 41.00] | 33.00 [27.00, 40.00] |
| Missing | 74 (4.3) | 13 (4.1) | 13 (4.5) | 27 (4.3) | 21 (4.2) |
| Age at diagnosis, median [IQR] | 35.00 [28.00, 43.00] | 32.00 [26.00, 38.00] | 34.00 [27.00, 41.00] | 36.00 [28.00, 44.75] | 35.00 [29.00, 43.00] |
| Age at first DMT, median [IQR] | 35.00 [28.00, 43.00] | 34.00 [27.00, 42.00] | 35.50 [28.00, 42.00] | 36.00 [29.00, 45.00] | 35.00 [29.00, 43.00] |
| Not started DMT in observation  period | 80 (4.6) | 26 (8.3) | 6 (2.1) | 21 (3.3) | 27 (5.4) |
| Initiated DMT ≥ 12 months after   diagnosis | 212/1658 (12.8) | 93/1658 (32.3) | 51/1658 (18.0) | 53/1658 (8.6) | 15/1658 (3.2) |
| Initiated DMT ≥ 24 months after   diagnosis | 139/1658 (8.4) | 71/1658 (24.7) | 36/1658 (12.7) | 27/1658 (4.4) | 5/1658 (1.1) |
| MS type, N (%) |  |  |  |  |  |
| CIS | 95 (5.5) | 17 (5.4) | 8 (2.8) | 39 (6.2) | 31 (6.2) |
| RRMS | 1643 (94.5) | 298 (94.6) | 282 (97.2) | 595 (93.8) | 469 (93.8) |
| First DMT Category, N (%) |  |  |  |  |  |
| High | 186 (10.7) | 6 (1.9) | 15 (5.2) | 36 (5.7) | 129 (25.8) |
| Moderate | 541 (31.1) | 12 (3.8) | 11 (3.8) | 256 (40.4) | 262 (52.4) |
| Low | 931 (53.6) | 270 (86.0) | 258 (89.0) | 321 (50.6) | 82 (16.4) |
| Untreated | 80 (4.6) | 26 (8.3) | 6 (2.1) | 21 (3.3) | 27 (5.4) |
| Comorbidities, N (%) |  |  |  |  |  |
| No | 710 (40.9) | 116 (36.8) | 107 (36.9) | 254 (40.1) | 233 (46.7) |
| Yes (≥1) | 658 (37.9) | 134 (42.5) | 123 (42.4) | 236 (37.2) | 166 (33.2) |
| Missing | 370 (21.3) | 65 (20.6) | 60 (20.7) | 144 (22.7) | 101 (20.2) |
| Diagnosis setting, N (%) |  |  |  |  |  |
| General practitioner | 20 (1.2) | 4 (1.3) | 4 (1.4) | 8 (1.3) | 4 (0.8) |
| Neurological practice | 659 (37.9) | 154 (48.9) | 113 (39.0) | 262 (41.3) | 131 (26.2) |
| Hospital | 844 (48.6) | 137 (43.5) | 150 (51.7) | 329 (51.9) | 228 (45.6) |
| Other | 12 (0.7) | 4 (1.3) | 2 (0.7) | 4 (0.6) | 2 (0.4) |
| Missing | 203 (11.7) | 16 (5.1) | 21 (7.2) | 31 (4.9) | 135 (27.0) |

**Supplementary Table 2**: Demographic and health-related characteristics of the SMSR study population used for the time to first DMT analysis overall and stratified by diagnosis period. MS: multiple sclerosis; CIS: clinically isolated syndrome; RRMS: relapsing-remitting MS.


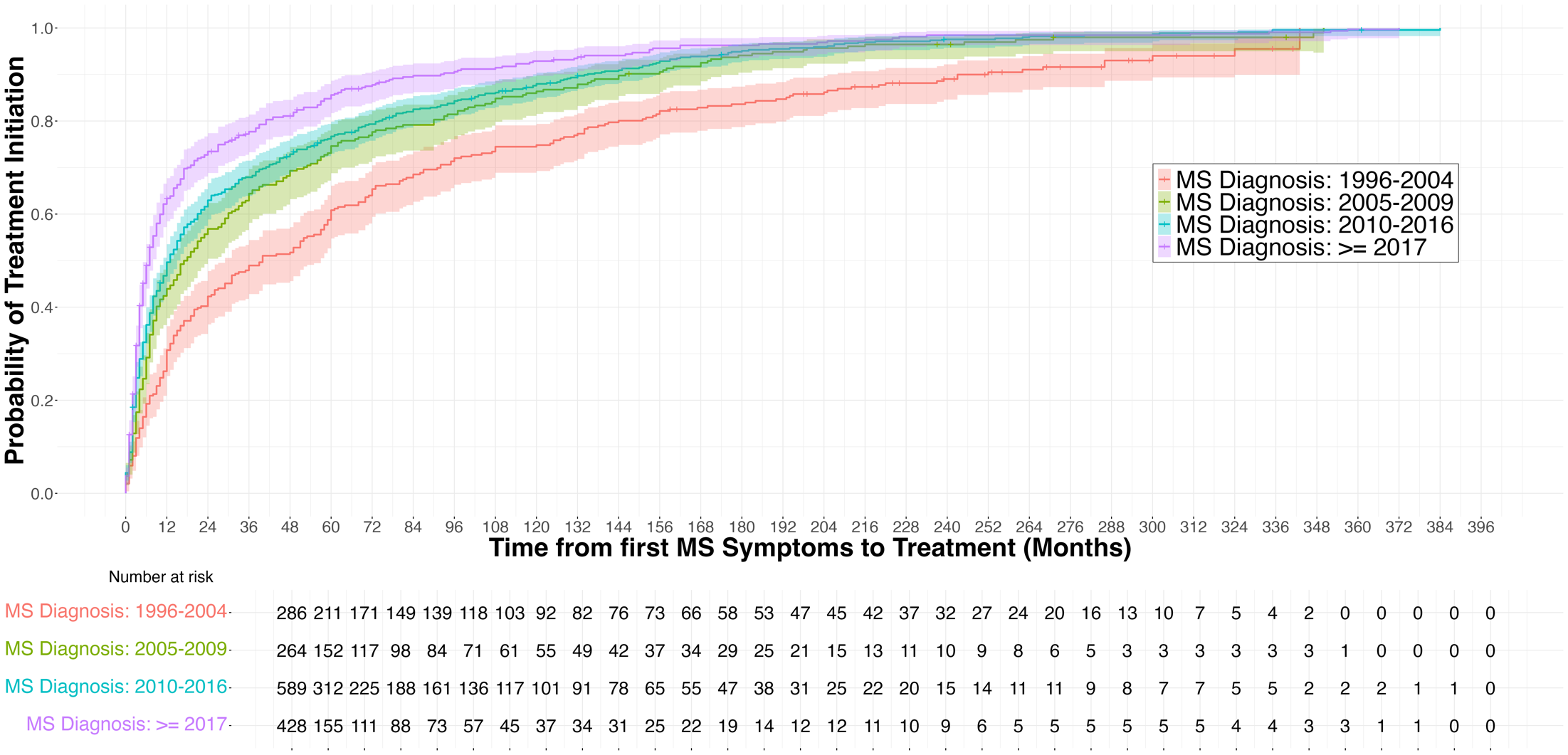


**Supplementary Figure 1**: Time from first MS symptoms to first disease-modifying therapy (DMT) by diagnosis period. The failure curves, derived from Kaplan-Meier analysis, display the cumulative probability of treatment initiation over time since the first MS symptoms, stratified by diagnosis period: 1995-2004, 2005-2009, 2010-2016, and $\geq$2017. Shaded areas represent 95% confidence intervals. This analysis included fewer participants due to missing data on symptom onset (N=1,567 vs. 1,738 in the main analysis). MS: multiple sclerosis.

**Supplementary Figure 2**: Temporal trends in the uptake of disease-modifying therapies (DMTs) for multiple sclerosis (MS) by efficacy category from 1995-2024. Each colored line represents the annual proportion of total DMT initiations, stratified by individuals DMTs. DMT: disease-modifying therapy.


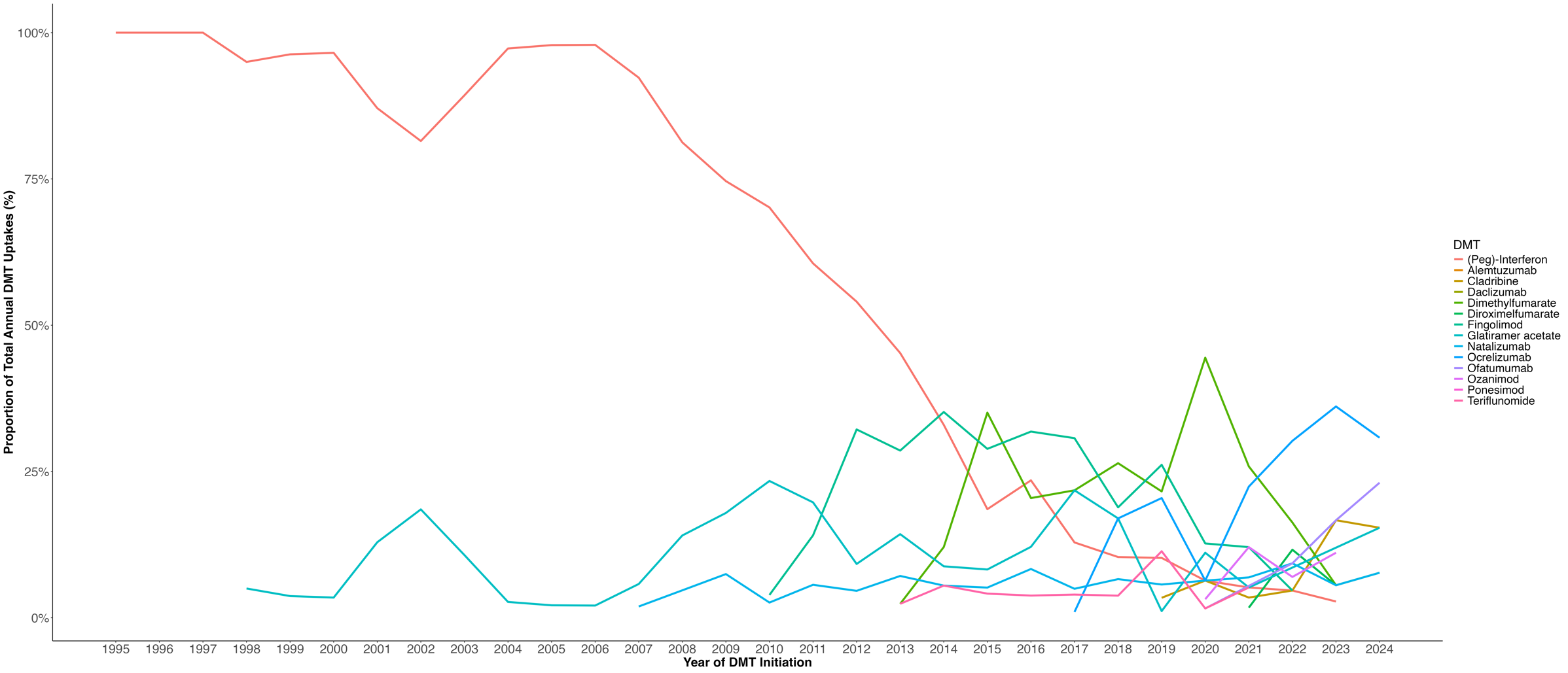


**Supplementary Table 1**: Demographic and health-related characteristics of the SMSR study sample included in the analysis. Numbers are shown overall and stratified by diagnosis period.

|  |  | | **DMT Initiation Year** | | | | | | | | | | | | | | | | | | | | | | | | | | | |  |
| --- | --- | --- | --- | --- | --- | --- | --- | --- | --- | --- | --- | --- | --- | --- | --- | --- | --- | --- | --- | --- | --- | --- | --- | --- | --- | --- | --- | --- | --- | --- | --- |
|  | **1995** | **1996** | | **1997** | **1998** | **1999** | **2000** | **2001** | **2002** | **2003** | **2004** | **2005** | **2006** | **2007** | **2008** | **2009** | **2010** | **2011** | **2012** | **2013** | **2014** | **2015** | **2016** | **2017** | **2018** | **2019** | **2020** | **2021** | **2022** | **2023** | **2024** |
| **N** | 8 | 6 | | 8 | 20 | 27 | 29 | 31 | 27 | 28 | 37 | 47 | 51 | 53 | 64 | 67 | 77 | 72 | 87 | 86 | 91 | 97 | 132 | 104 | 106 | 89 | 63 | 58 | 43 | 37 | 13 |
| **Low efficacy (%)** |  |  | |  |  |  |  |  |  |  |  |  |  |  |  |  |  |  |  |  |  |  |  |  |  |  |  |  |  |  |  |
| Beta interferons | 100 | 100 | | 100 | 95.0 | 96.3 | 96.6 | 87.1 | 81.5 | 89.3 | 97.3 | 97.9 | 92.2 | 90.6 | 81.2 | 74.6 | 70.1 | 60.6 | 54.0 | 44.2 | 33.0 | 18.6 | 23.5 | 12.5 | 10.4 | 10.1 | 6.4 | 5.2 | 4.7 | 2.7 | 0.0 |
| Glatiramer acetate | 0.0 | 0.0 | | 0.0 | 5.0 | 3.7 | 3.4 | 12.9 | 18.5 | 10.7 | 2.7 | 2.1 | 2.0 | 5.7 | 14.1 | 17.9 | 23.4 | 19.7 | 9.2 | 13.9 | 8.8 | 8.2 | 12.1 | 21.2 | 17.0 | 1.3 | 11.1 | 5.2 | 0.0 | 0.0 | 15.4 |
| **Moderate efficacy (%)** |  |  | |  |  |  |  |  |  |  |  |  |  |  |  |  |  |  |  |  |  |  |  |  |  |  |  |  |  |  |  |
| Fingolimod | 0.0 | 0.0 | | 0.0 | 0.0 | 0.0 | 0.0 | 0.0 | 0.0 | 0.0 | 0.0 | 0.0 | 0.0 | 0.0 | 0.0 | 0.0 | 3.9 | 14.1 | 32.2 | 27.9 | 35.2 | 28.9 | 31.8 | 29.8 | 18.9 | 25.8 | 12.7 | 12.1 | 4.7 | 0.0 | 0.0 |
| Dimethyl fumarate | 0.0 | 0.0 | | 0.0 | 0.0 | 0.0 | 0.0 | 0.0 | 0.0 | 0.0 | 0.0 | 0.0 | 0.0 | 0.0 | 0.0 | 0.0 | 0.0 | 0.0 | 0.0 | 3.5 | 12.0 | 35.0 | 20.5 | 21.1 | 26.4 | 21.3 | 44.4 | 25.9 | 16.2 | 5.5 | 7.7 |
| Diroximelfumarate | 0.0 | 0.0 | | 0.0 | 0.0 | 0.0 | 0.0 | 0.0 | 0.0 | 0.0 | 0.0 | 0.0 | 0.0 | 0.0 | 0.0 | 0.0 | 0.0 | 0.0 | 0.0 | 0.0 | 0.0 | 0.0 | 0.0 | 0.0 | 0.0 | 0.0 | 0.0 | 1.7 | 11.6 | 5.4 | 0.0 |
| Ozanimod | 0.0 | 0.0 | | 0.0 | 0.0 | 0.0 | 0.0 | 0.0 | 0.0 | 0.0 | 0.0 | 0.0 | 0.0 | 0.0 | 0.0 | 0.0 | 0.0 | 0.0 | 0.0 | 0.0 | 0.0 | 0.0 | 0.0 | 0.0 | 0.0 | 0.0 | 3.2 | 12.0 | 7.0 | 13.5 | 0.0 |
| Daclizumab | 0.0 | 0.0 | | 0.0 | 0.0 | 0.0 | 0.0 | 0.0 | 0.0 | 0.0 | 0.0 | 0.0 | 0.0 | 0.0 | 0.0 | 0.0 | 0.0 | 0.0 | 0.0 | 0.0 | 0.0 | 0.0 | 0.0 | 1.9 | 0.0 | 0.0 | 0.0 | 0.0 | 0.0 | 0.0 | 0.0 |
| Ponesimod | 0.0 | 0.0 | | 0.0 | 0.0 | 0.0 | 0.0 | 0.0 | 0.0 | 0.0 | 0.0 | 0.0 | 0.0 | 0.0 | 0.0 | 0.0 | 0.0 | 0.0 | 0.0 | 0.0 | 0.0 | 0.0 | 0.0 | 0.0 | 0.0 | 0.0 | 0.0 | 0.0 | 2.3 | 0.0 | 0.0 |
| Teriflunomide | 0.0 | 0.0 | | 0.0 | 0.0 | 0.0 | 0.0 | 0.0 | 0.0 | 0.0 | 0.0 | 0.0 | 0.0 | 0.0 | 0.0 | 0.0 | 0.0 | 0.0 | 0.0 | 3.5 | 5.5 | 4.1 | 3.8 | 3.8 | 3.8 | 11.2 | 1.6 | 5.2 | 0.0 | 0.0 | 0.0 |
| Cladribine | 0.0 | 0.0 | | 0.0 | 0.0 | 0.0 | 0.0 | 0.0 | 0.0 | 0.0 | 0.0 | 0.0 | 0.0 | 0.0 | 0.0 | 0.0 | 0.0 | 0.0 | 0.0 | 0.0 | 0.0 | 0.0 | 0.0 | 0.0 | 0.0 | 3.4 | 6.3 | 3.4 | 4.7 | 16.2 | 15.4 |
| **High efficacy (%)** |  |  | |  |  |  |  |  |  |  |  |  |  |  |  |  |  |  |  |  |  |  |  |  |  |  |  |  |  |  |  |
| Ocrelizumab | 0.0 | 0.0 | | 0.0 | 0.0 | 0.0 | 0.0 | 0.0 | 0.0 | 0.0 | 0.0 | 0.0 | 0.0 | 0.0 | 0.0 | 0.0 | 0.0 | 0.0 | 0.0 | 0.0 | 0.0 | 0.0 | 0.0 | 1.0 | 16.9 | 20.2 | 6.3 | 22.4 | 30.2 | 35.1 | 30.8 |
| Natalizumab | 0.0 | 0.0 | | 0.0 | 0.0 | 0.0 | 0.0 | 0.0 | 0.0 | 0.0 | 0.0 | 0.0 | 5.8 | 3.7 | 4.7 | 7.5 | 2.6 | 5.6 | 4.6 | 7.0 | 5.5 | 5.2 | 8.3 | 4.8 | 6.6 | 5.6 | 6.3 | 6.9 | 9.3 | 5.4 | 7.6 |
| Alemtuzumab | 0.0 | 0.0 | | 0.0 | 0.0 | 0.0 | 0.0 | 0.0 | 0.0 | 0.0 | 0.0 | 0.0 | 0.0 | 0.0 | 0.0 | 0.0 | 0.0 | 0.0 | 0.0 | 0.0 | 0.0 | 0.0 | 0.0 | 1.0 | 0.0 | 0.0 | 0.0 | 0.0 | 0.0 | 0.0 | 0.0 |
| Ofatumumab | 0.0 | 0.0 | | 0.0 | 0.0 | 0.0 | 0.0 | 0.0 | 0.0 | 0.0 | 0.0 | 0.0 | 0.0 | 0.0 | 0.0 | 0.0 | 0.0 | 0.0 | 0.0 | 0.0 | 0.0 | 0.0 | 0.0 | 0.0 | 0.0 | 0.0 | 1.6 | 0.0 | 9.3 | 16.2 | 23.1 |
| Rituximab | 0.0 | 0.0 | | 0.0 | 0.0 | 0.0 | 0.0 | 0.0 | 0.0 | 0.0 | 0.0 | 0.0 | 0.0 | 0.0 | 0.0 | 0.0 | 0.0 | 0.0 | 0.0 | 0.0 | 0.0 | 0.0 | 0.0 | 2.9 | 0.0 | 1.1 | 0.0 | 0.0 | 0.0 | 0.0 | 0.0 |

**Supplementary Table 3**: Annual counts and percentages of disease-modifying therapy (DMT) initiations in the Swiss Multiple Sclerosis Registry (SMSR) study population for the time period 1995-2024. N indicates the total number of DMT initiations per year; percentages reflect the proportion of those initiations attributable to each individual DMT, grouped by low-, moderate- and high-efficacy DMTs. DMT: Disease-modifying therapy.

|  |  |  |  |  |  |  |  |  |  |  |
| --- | --- | --- | --- | --- | --- | --- | --- | --- | --- | --- |
|  | **Interferons** | | **Glatiramer acetate** | **Fumarates** | **Teriflunomide** | **S1P modulators** | **Cladribine** | **Anti-CD20** | **Natalizumab** |  |
| **N** | 108 | | 54 | 139 | 23 | 170 | 18 | 86 | 51 |  |
| **Suspected ADRs, N (%)** |  | |  |  |  |  |  |  |  |  |
| *Non-severe suspected ADR* |  | |  |  |  |  |  |  |  |  |
| Pain | 1 (0.9) | | 1 (1.9) | 0 (0.0) | 0 (0.0) | 0 (0.0) | 0 (0.0) | 0 (0.0) | 0 (0.0) |  |
| Flu-like symptoms | 71 (65.7) | | 7 (13.0) | 14 (10.1) | 3 (13.0) | 14 (8.2) | 3 (16.7) | 14 (16.3) | 6 (11.8) |  |
| Headache | 48 (44.4) | | 10 (18.5) | 12 (8.6) | 6 (26.1) | 16 (9.4) | 2 (11.1) | 13 (15.1) | 4 (7.8) |  |
| Gastrointestinal problems | 6 (5.6) | | 4 (7.4) | 26 (18.7) | 1 (4.3) | 6 (3.5) | 3 (16.7) | 2 (2.3) | 1 (2.0) |  |
| Sleep problems | 22 (20.4) | | 8 (14.8) | 12 (8.6) | 4 (17.4) | 12 (7.1) | 2 (11.1) | 5 (5.8) | 6 (11.8) |  |
| Fatigue | 35 (32.4) | | 15 (27.8) | 29 (20.9) | 6 (26.1) | 23 (13.5) | 6 (33.3) | 17 (19.8) | 5 (9.8) |  |
| Skin problems | 1 (0.9) | | 1 (1.9) | 1 (0.7) | 0 (0.0) | 0 (0.0) | 0 (0.0) | 0 (0.0) | 0 (0.0) |  |
| Allergic reactions | 0 (0.0) | | 3 (5.6) | 3 (2.2) | 0 (0.0) | 1 (0.6) | 0 (0.0) | 3 (3.5) | 0 (0.0) |  |
| Hot Flushes | 25 (23.1) | | 11 (20.4) | 72 (51.8) | 2 (8.7) | 11 (6.5) | 2 (11.1) | 13 (15.1) | 3 (5.9) |  |
| Hair loss | 10 (9.3) | | 7 (13.0) | 17 (12.2) | 9 (39.1) | 11 (6.5) | 1 (5.6) | 4 (4.7) | 2 (3.9) |  |
| Dizziness | 1 (0.9) | | 2 (3.7) | 3 (2.2) | 0 (0.0) | 3 (1.8) | 0 (0.0) | 1 (1.2) | 2 (3.9) |  |
| Neuropsychiatric symptoms | 9 (8.3) | | 6 (11.1) | 20 (14.4) | 1 (4.3) | 4 (2.4) | 2 (11.1) | 5 (5.8) | 1 (2.0) |  |
| *Severe suspected ADR* |  | |  |  |  |  |  |  |  |  |
| Hepatotoxicity | 0 (0.0) | | 0 (0.0) | 0 (0.0) | 0 (0.0) | 1 (0.6) | 0 (0.0) | 0 (0.0) | 0 (0.0) |  |
| PML | 0 (0.0) | | 0 (0.0) | 0 (0.0) | 0 (0.0) | 2 (1.2) | 0 (0.0) | 0 (0.0) | 2 (3.9) |  |
| Opportunistic infections | 1 (0.9) | | 0 (0.0) | 4 (2.9) | 0 (0.0) | 5 (2.9) | 2 (11.1) | 7 (8.1) | 2 (3.9) |  |
| Depression | 8 (7.4) | | 3 (5.6) | 8 (5.8) | 2 (8.7) | 6 (3.5) | 1 (5.6) | 1 (1.2) | 4 (7.8) |  |
| Reported any severe suspected ADR, N (%) | 8 (7.4) | | 3 (5.6) | 10 (7.2) | 2 (8.7) | 11 (6.5) | 2 (11.1) | 7 (8.1) | 6 (11.8) |  |
|  |  | |  |  |  |  |  |  |  |  |
|  |  | |  |  |  |  |  |  |  |  |

**Supplementary Table 4**: Frequency or reported suspected adverse drug reaction (ADRs) stratified by disease-modifying therapy (DMT) pharmacological class. Interferons include (peg-)interferon beta-1a/1b; Fumarates include dimethyl fumarate and diroximel fumarate; S1P modulators include fingolimod, ozanimod and ponesimod; Anti-CD20 include ocrelizumab, ofatumumab and rituximab. PML: progressive multifocal leukoencephalopathy. ADR: adverse drug reaction.
