# Supplementary material for: From diagnosis to treatment: patterns in disease-modifying therapy initiation in multiple sclerosis
Source: Ther Adv Neurol Disord. 2025 Dec 5;18:17562864251398472. doi: 10.1177/17562864251398472 (PMC12681631; doi:10.1177/17562864251398472)
Supplement: sj-docx-2-tan-10.1177_17562864251398472 – Supplemental material for From diagnosis to treatment: patterns in disease-modifying therapy initiation in multiple sclerosis [file sj-docx-2-tan-10.1177_17562864251398472.docx]

**Main Survey**

**MS REGISTRY**

**
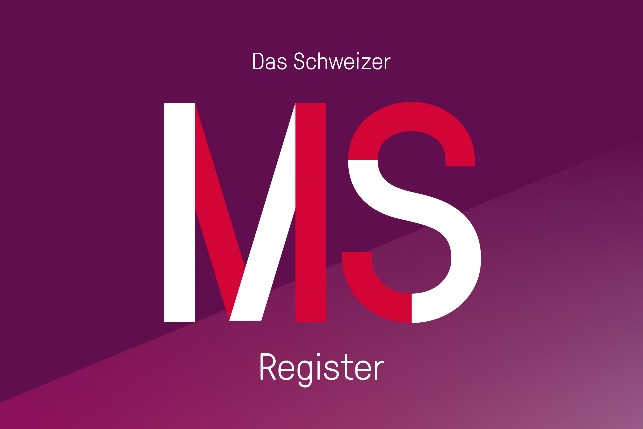
**

**Swiss MS Registry**

**2022**

**Part A: Family circumstances and living situation**

***Below you will find some questions about your family circumstances*** *(including your own children and your partner’s children)* ***as well as your living situation.***

|  | |
| --- | --- |
| **A1.** | **Which of the following best describes your current living situation?**  *Please select only one of the following answers:* |
|  |  |
| O | With husband / wife / partner |
| O | With family |
| O | Living alone or as a single parent |
| O | With parents |
| O | With colleagues(s) / friends / relatives / in a shared apartment |
| O | Clinic / residential home / therapeutic residential community |
|  | *If clinic / residential home / therapeutic residential community, please specify (name)?* |
|  |  |
|  | ----------------------------------------------------- |
| O | Other |
|  | *If other living situation, please specify:* |
|  | *------------------------------------------------------* |
|  | |

| **A2.** | **How many children do you have (including the children of your partner, adopted and foster children)?**  *Please specify how many.*   \| 1 \|  \| \| --- \| --- \|   E.g. |
| --- | --- | --- | --- |

|  |  |
| --- | --- |

|  |
| --- |

|  |
| --- |

| 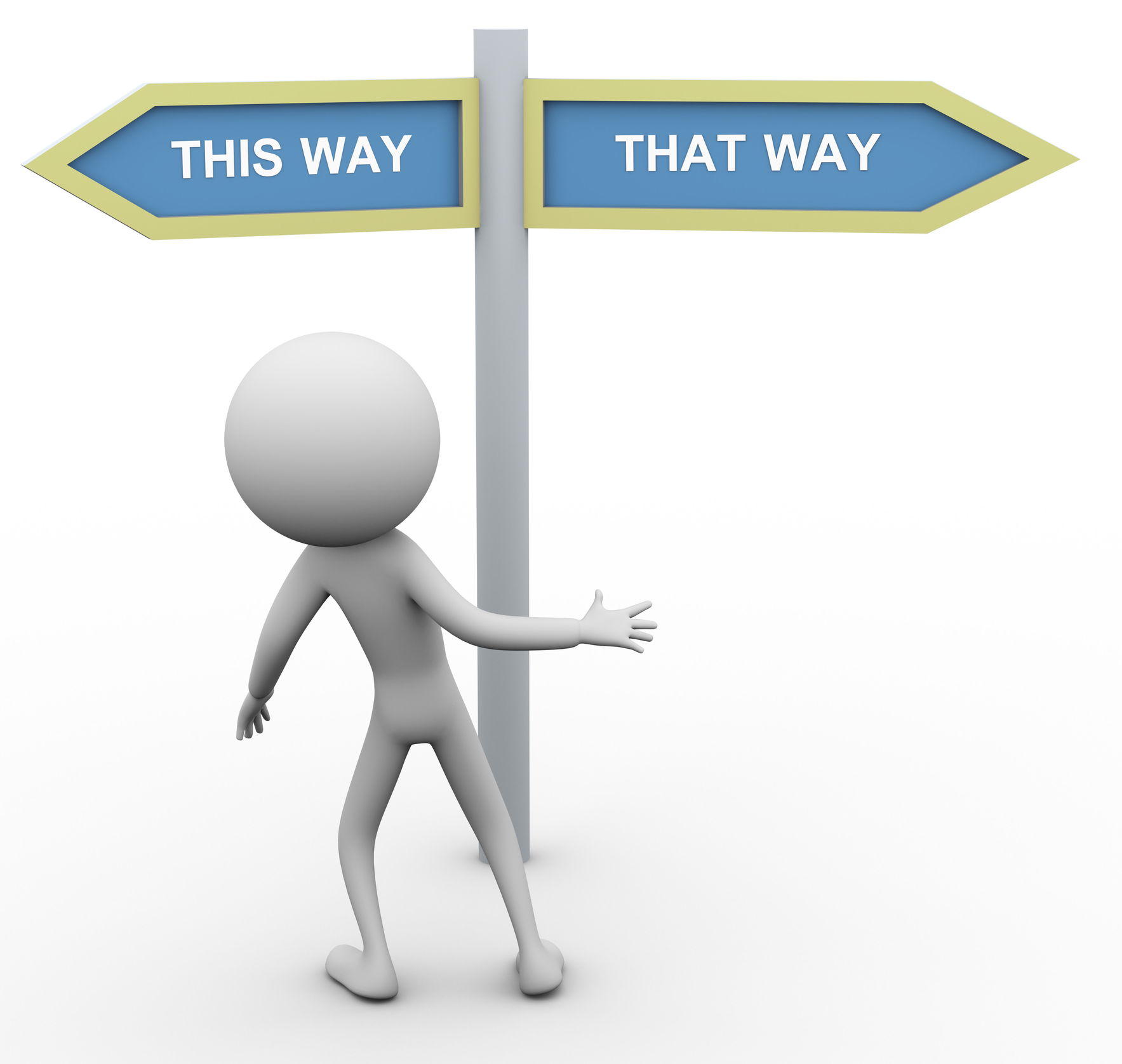 | *The following question A3 must* ***only be answered by persons*** ***who*** ***have children****, i.e., who answered at least 1 to the previous question*  ***Otherwise*** *(i.e. all others): Please continue with question A4 (Page 5).* |
| --- | --- |

| **A3.** | **Please enter the year of birth of your child/children.**  *Only numbers may be entered in these fields.*   \| *E.g.* 1. Child \| 2 \| 0 \| 0 \| 2 \| Year \| \| --- \| --- \| --- \| --- \| --- \| --- \| |
| --- | --- | --- | --- | --- | --- | --- | --- |
| \| 1. Child \|  \|  \|  \|  \| Year \| \| --- \| --- \| --- \| --- \| --- \| --- \| \| 2. Child \|  \|  \|  \|  \| Year \| \| 3. Child \|  \|  \|  \|  \| Year \| \| 4. Child \|  \|  \|  \|  \| Year \| \| 5. Child \|  \|  \|  \|  \| Year \| \| 6. Child \|  \|  \|  \|  \| Year \| \| 7. Child \|  \|  \|  \|  \| Year \| \| 8. Child \|  \|  \|  \|  \| Year \| \| 9. Child \|  \|  \|  \|  \| Year \| \| 10. Child \|  \|  \|  \|  \| Year \| | |

|  |
| --- |

| **A4.** | **What is your current marital status?** |
| --- | --- |

| O | | Single | |
| --- | --- | --- | --- |
| O | | Married | |
| O | | Registered partnership | |
| O | | Separated | |
| O | | Divorced | |
| O | | Widowed | |
|  | |  |  |

**Part B: Education / Occupation**

*The following section contains some questions about your education and your occupation.*

|  | |
| --- | --- |
| **B1.** | **What is your highest level of education?**  *Please select only one of the following answers:* |
|  |  |
| O | Compulsory schooling not completed (or up to and including 7^th^ grade) |
| O | Compulsory schooling completed |
| O | Vocational training / Apprenticeship |
| O | Matura, Gymnasium, Vocational baccalaureate, specialized secondary school |
| O | Advanced vocational education (e.g., technical school, master craftsman training) |
| O | University degree (technical college or university) |
| O | Other |
|  | *If other type of education, please specify?* |
|  | *------------------------------------------------* |
|  | |

|  | | |
| --- | --- | --- |
| **B2.** | **Are you currently employed?**  *Please select only one of the following answers.* | |
|  |  | |
| O | Yes | |
| O | No | |
|  | |  |

| 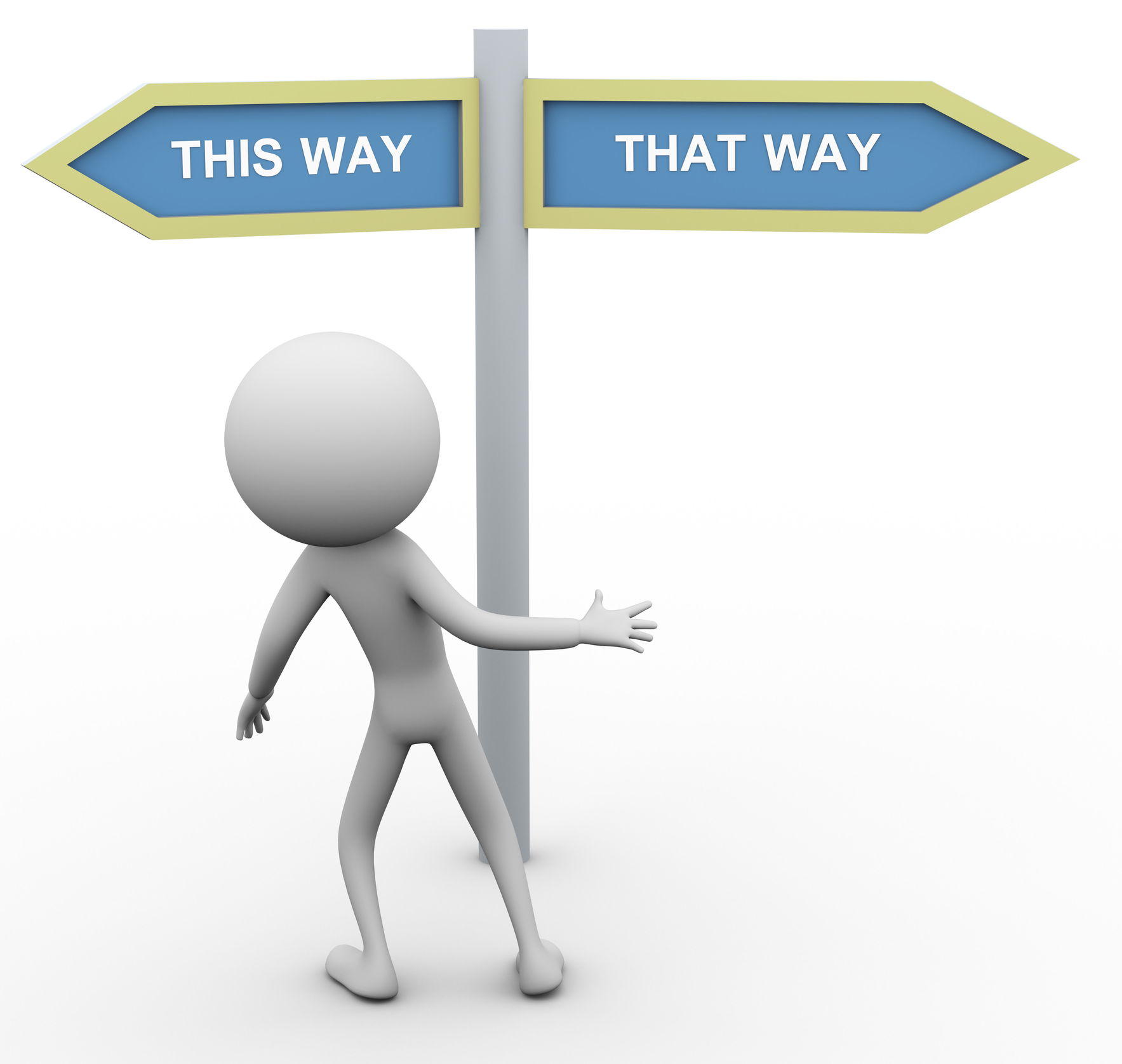 | *If* ***yes***  *--> continue with question* ***B3****.*  *If* ***no*** *--> continue with question* ***B4*** *(Page 8).* |
| --- | --- |

|  | |
| --- | --- |
| **B3.** | **What is your current level of employment (in percent)?**  *Please enter the percentage.*  *E.g.*   \|  \| 4 \| 0 \| % \| \| --- \| --- \| --- \| --- \| |

|  |  |  | % |
| --- | --- | --- | --- |

|  |
| --- |

|  | | |
| --- | --- | --- |
| **B4.** | **Do you receive benefits from the disability insurance (IV)?**  *Please select only one of the following answers.* | |
|  |  | |
| O | Yes | |
| O | No | |
| O | Have applied for benefits | |
|  | |  |

| 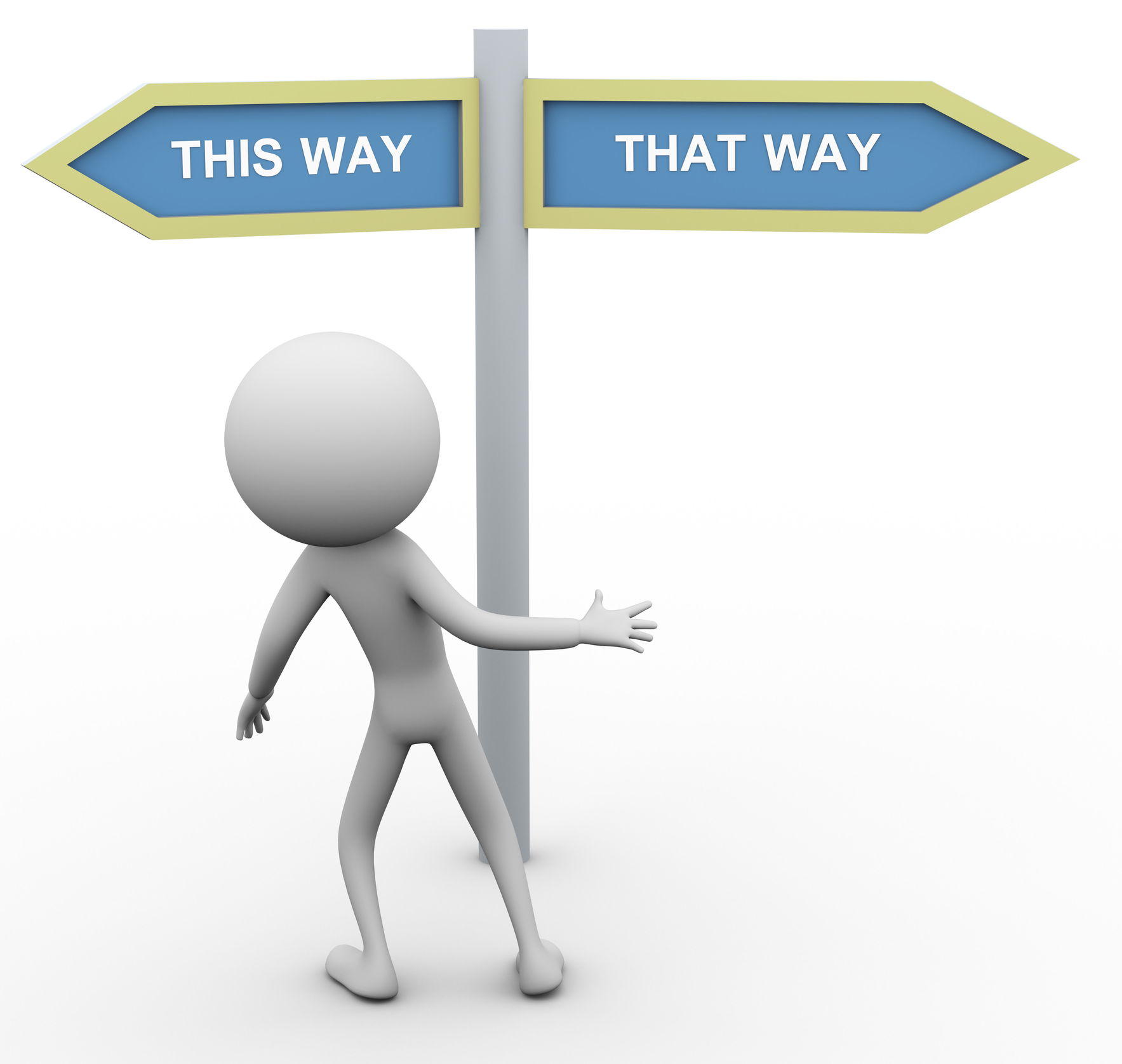 | *If* ***yes*** *--> continue with question* ***B5****.*  *If* ***no*** *--> continue with question* ***C1*** *(Page 9).* |
| --- | --- |

|  | |
| --- | --- |
| **B5.** | **What percentage of disability benefits are you entitled to?**  *Please enter the percentage.*   \|  \| 5 \| 0 \| % \| \| --- \| --- \| --- \| --- \|   *E.g.* |

|  |  |  | % |
| --- | --- | --- | --- |

|  |
| --- |

**Part C: Social support with household chores / medical care**

*Below are some questions about social support with household chores and medical care.*

| **C1.** | **Are you able to do household chores independently?**  *Please select only one of the following answers.* |
| --- | --- |
|  |  |
| O | Yes, all of them |
| O | Most of them |
| O | Some of them |
| O | Few of them |
| O | No, none of them |
|  |  |

*Comments:*

|  |
| --- |

|  | | |
| --- | --- | --- |
| **C2.** | **Do you currently receive assistance with household tasks?**  *Please select only one of the following answers.* | |
|  |  | |
| O | Yes | |
| O | No | |
|  | |  |

| 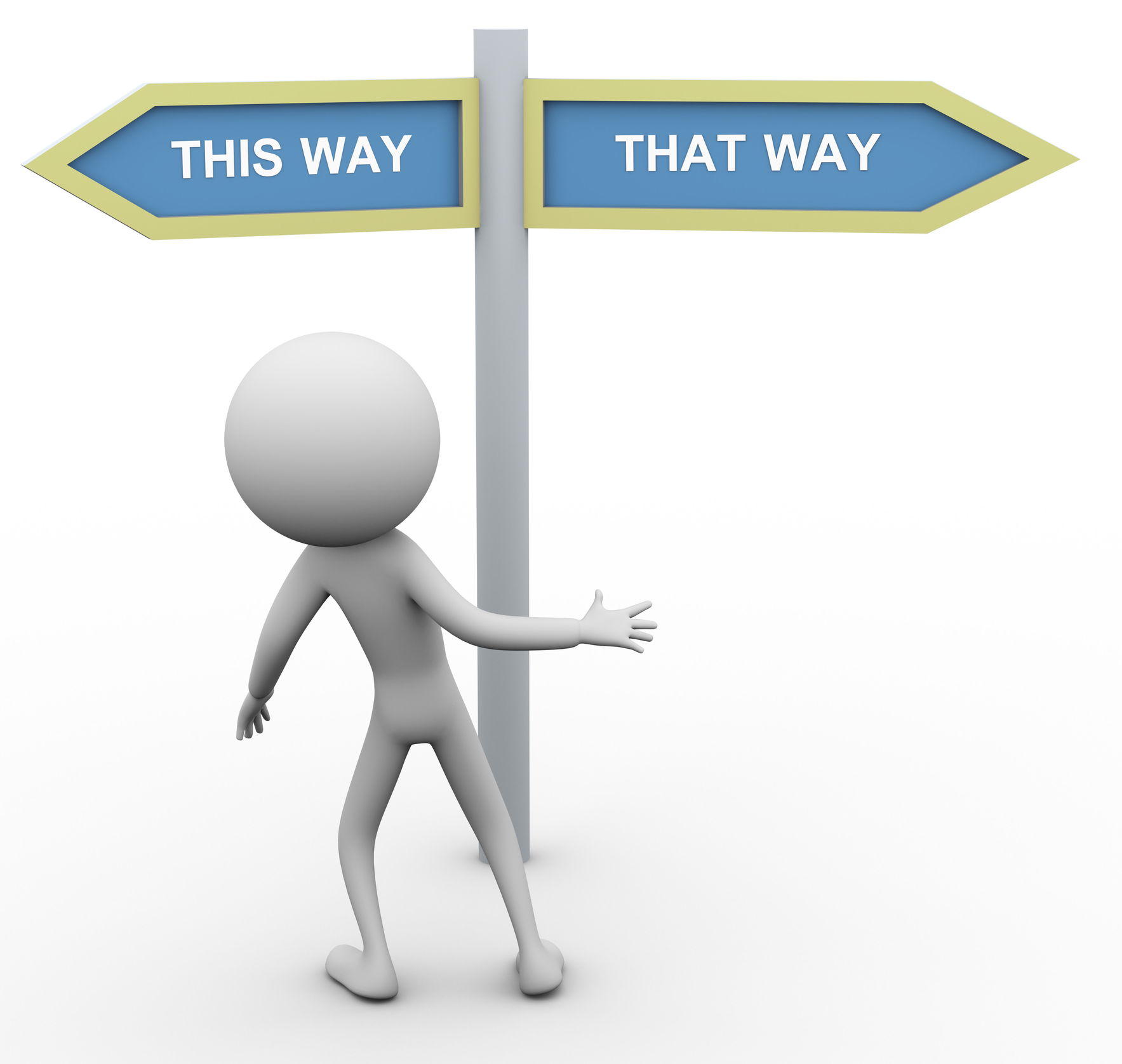 | *If* ***yes*** *--> continue with question* ***C3****.*  *If* ***no*** *--> continue with question* ***C4*** *(page 12).* |
| --- | --- |

|  | | |
| --- | --- | --- |
| **C3.** | | **Please indicate who provides you with this assistance.**  *Please select all applicable answers.* |
|  | |  |
| O | | Family members, partner |
| O | | Voluntary help from friends acquaintances, neighbors |
| O | | Paid professional help (e.g., domestic help, Spitex (home care), meal service) |
| O | | Other |
|  | | *If other support, what kind?* |
|  | | *----------------------------------------------------* |
|  | |  |

|  | |
| --- | --- |
| **C4.** | **Which of the following types of medical care are you currently receiving?**  *Please select all applicable answers.* |
|  |  |
| O | None |
| O | Home care |
| O | Outpatient care |
| O | Short-term care |
| O | Day care |
| O | In-patient care |
| O | Other |
|  | *If other types of care, which?*  *---------------------------------------------------* |
|  |  |
|  | |

| **C5.** | **Which of the following specialists do you consult regularly?**  *Please select the applicable answer for each item.* |
| --- | --- |
|  |  |

|  | | |  | | **Yes** | |  | | **No** | |  | |  | |  |
| --- | --- | --- | --- | --- | --- | --- | --- | --- | --- | --- | --- | --- | --- | --- | --- |
|  | | |  |  | |  | |  | |  | |  | |  | |
|  | | Family doctor |  | O | |  | | O | |  | |  | |  | |
|  | | Neurologist at the clinic |  | O | |  | | O | |  | |  | |  | |
|  | | Private neurologist |  | O | |  | | O | |  | |  | |  | |
|  | | Physiotherapist specializing in neurology |  | O | |  | | O | |  | |  | |  | |
|  | |  |  |  | |  | |  | |  | |  | |  | |
|  | | Physiotherapist with a general focus |  | O | |  | | O | |  | |  | |  | |
|  | | Psychologist / Psychiatrist |  | O | |  | | O | |  | |  | |  | |
|  | | Osteopath |  | O | |  | | O | |  | |  | |  | |
|  | | Naturopath |  | O | |  | | O | |  | |  | |  | |
|  | | Acupuncture specialist |  | O | |  | | O | |  | |  | |  | |
|  | | TCM specialist |  | O | |  | | O | |  | |  | |  | |
|  | | Other |  | O | |  | | O | |  | |  | |  | |
|  | | *If another specialist, which one?* |  |  | |  | |  | |  | |  | |  | |
|  | |  |  |  | |  | |  | |  | |  | |  | |
|  | | *----------------------------------------------* |  |  | |  | |  | |  | |  | |  | |
|  | | | | | | | | | | | | | | | |

|  | |
| --- | --- |
| **C6.** | **What medical aids are you currently using?**  *Please select all applicable answers.* |
|  |  |
| O | None |
| O | Walking aid, Cane |
| O | Bath entry aid |
| O | Wheelchair |
| O | Walker |
| O | Joint supports |
| O | Arm splint |
| O | Handle grip |
| O | Waterproof cover |
| O | Other |
|  | *If other aids, which ones?* |
|  | *----------------------------------------------* |
|  | |

|  | |
| --- | --- |
| **C7.** | **With whom do you talk openly about your MS?**  *Please select all applicable answers.* |
|  |  |
| O | Husband / Wife / Partner |
| O | Family / Relatives |
| O | Friends, Colleagues |
| O | Neighbors |
| O | Leisure contacts |
| O | Other people with MS, MS groups, etc. |
| O | Work colleagues |
| O | Supervisors |
| O | No one |
| O | Other |
|  | *If other people, who?* |
|  | *-----------------------------------------* |
|  | |

**Part D: MS disease progression**

*Below are some questions about disease progression, relapses, and the progression of MS.*

| **D1.** | **Please indicate the current type of progression of your MS disease.**  *Please select only one of the following answers.* |
| --- | --- |
|  |  |
| O | Clinically isolated syndrome (CIS) / no definitive MS diagnosis |
| O | Relapsing remitting MS (RRMS) |
| O | Primary progressive MS (PPMS) |
| O | Secondary progressive MS (SPMS) |
| O | Transition between two stages or other forms (please specify below) |
|  | *Any additional comments on your selection?* |
|  | |

|  |
| --- |

| **D2.** | **Which symptoms do you currently have?**  *Please select all applicable answers.* | | | | | |  |
| --- | --- | --- | --- | --- | --- | --- | --- |
|  | |  | |  |  |  |  |
| O | | None | | O | Balance disorders |  |  |
| O | | Visual disturbances | | O | Bladder disorders (e.g., weak bladder) |  |  |
| O | | Speech disorders | | O | Spasms (muscle cramps) |  |  |
| O | | Swallowing disorders (Dysphagia) | | O | Twitching, tics |  |  |
| O | | Weakness | | O | Tremor (shaking) |  |  |
| O | | Paralysis | | O | Bowel disorder (e.g., constipation) |  |  |
| O | | Tiredness, fatigue | | O | Epileptic seizures |  |  |
| O | | Sensory disturbances (e.g., numbness, tingling) | | O | Sexual dysfunction |  |  |
| O | | Dizziness | | O | Memory impairment |  |  |
| O | | Pain | | O | Depression |  |  |
| O | | Gait disorder | | O | Other |  |  |
|  | | | *If other symptoms, which ones?* | | | | |
|  | | | *-------------------------------------------* | | | | |
|  | | | | | | |  |

| 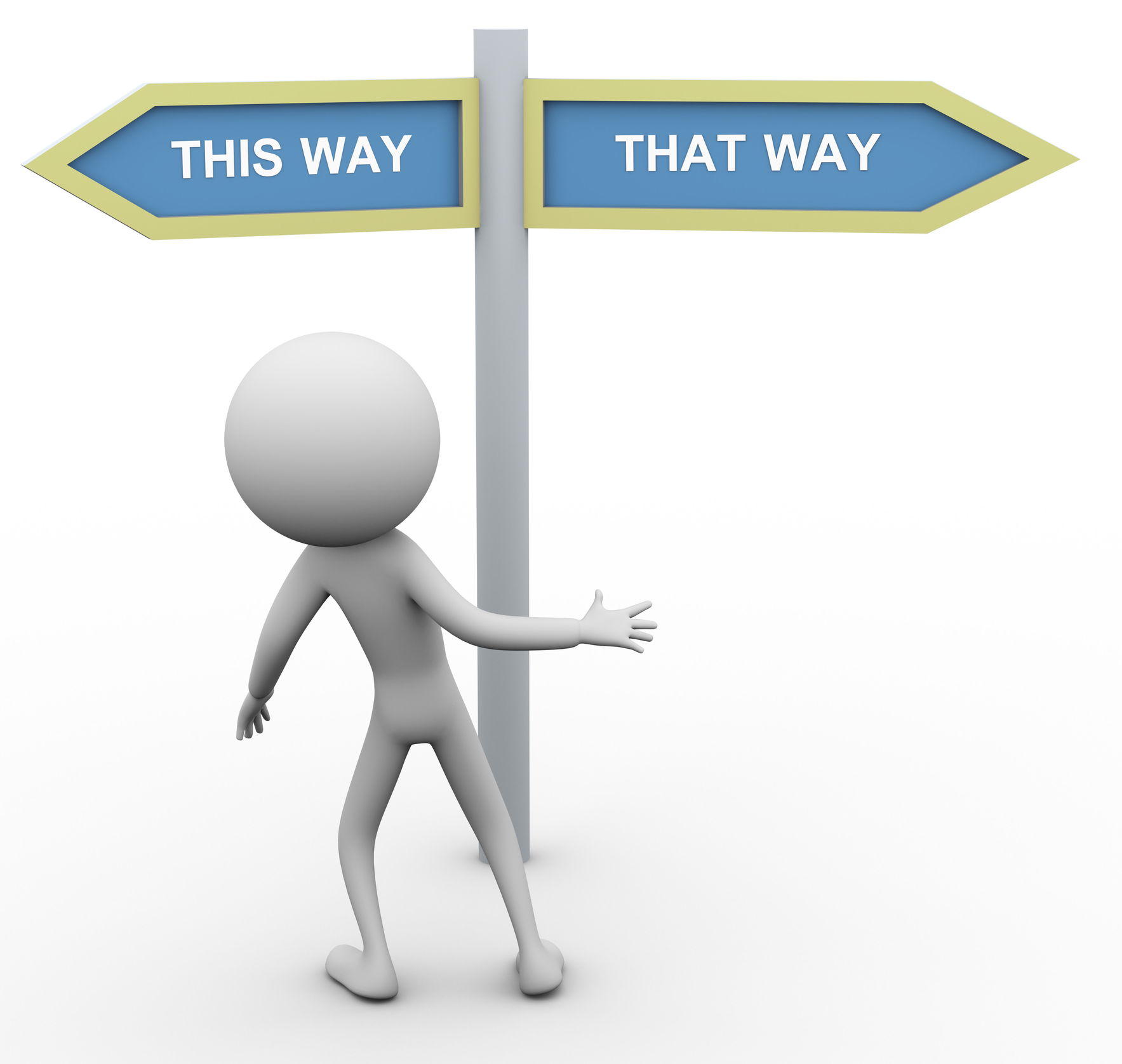 | *If you have* ***primary progressive MS*** *--> continue with question* ***E1*** *(Page 19).*  ***Other*** *--> continue with question* ***D3****.* |
| --- | --- |

| **D3.** | **Please indicate how many relapses you have had since the onset of the disease to date (including the first relapse).**  **If you cannot remember exactly or if there have been many relapses, round up to the nearest 10.** |
| --- | --- |
| \|  \|  \| Number of relapses \| \| --- \| --- \| --- \| | |

|  |
| --- |

| **D4.** | **When did you have your last relapse? Please indicate - if possible - the month and year.** |
| --- | --- |
| \|  \|  \| Month \|  \|  \|  \|  \|  \| Year \| \| --- \| --- \| --- \| --- \| --- \| --- \| --- \| --- \| --- \| \|  \|  \|  \|  \|  \|  \|  \|  \|  \| | |

|  |
| --- |

**Part E: Physical activity and exercise**

*Below are some questions about physical activity and exercise.*

|  | |
| --- | --- |
| **E1.** | **How far can you currently walk on flat ground?**  *Please select only one of the following answers.* |
|  |  |
| O | < 5 m |
| O | 5-10 m |
| O | 10-50 m |
| O | 50-100 m |
| O | 100-500 m |
| O | 500-1000 m |
| O | 1-5 km |
| O | > 5 km |
|  | |

|  | |
| --- | --- |
| **E2.** | **How many stairs can you currently manage?**  *Please select only one of the following answers.* |
|  |  |
| O | 0 |
| O | 1-20 (approx. 1 floor) |
| O | 21-50 (approx. 2-3 floors) |
| O | > 50 (more than 3 floors) |
|  | |

|  | |
| --- | --- |
| **E3.** | **Do you drive a car independently?**  *Please select only one of the following answers.* |
|  |  |
| O | Yes, with a standard car (without modifications) |
| O | Yes, with a modified car |
| O | No |
| O | No driver’s license |

|  |
| --- |

|  | |
| --- | --- |
| **E4.** | **Can you use public transportation independently?**  *Please select only one of the following answers.* |
|  |  |
| O | Yes |
| O | No |

|  |
| --- |

| **E5.** | **On how many days last week were you physically active for a total of 30 minutes or more, so that you had to breathe at least a little harder?**    *Examples of such activities include sports, exercise, training, brisk walking or cycling either for leisure or to get from place to place.*  *Please do not include physical activities carried out in the household or as part of your work.* |
| --- | --- |
|  |  |

Number of days last week:

|  |
| --- |

**Part F: Treatment**

*Below are some questions about MS treatments.*

| **F1.** | **Which of the following immunomodulatory drugs are you currently taking?**  *Please select all applicable answers.* | | | | | |  |
| --- | --- | --- | --- | --- | --- | --- | --- |
| O | | None | O | Imurek® / Azathioprin | |  |  |
| O | | Betaferon® / Interferon beta 1b | O | Synacthen® / Corticotropin | |  |  |
| O | | Extavia® / Interferon beta 1b | O | Sandimmun® / Cyclosporin | |  |  |
| O | | Avonex® / Interferon beta 1a | O | Endoxan® / Cyclophosphamid | |  |  |
| O | | Rebif® / Interferon beta 1a | O | MabThera® / Rituxan® / Rituximab | |  |  |
| O | | Plegridy® / Peginterferon beta 1a | O | Ocrevus® / Ocrelizumab | |  |  |
| O | | Copaxone® / Glatirameracetat | O | Mavenclad® / Cladribin | |  |  |
| O | | Gilenya® / Fingolimod | O | Mayzent® / Siponimod | |  |  |
| O | | Tysabri® / Natalizumab | O | Arzerra® / Kesimpta® / Ofatumumab | |  |  |
| O | | Tecfidera® / BG-12 / Dimethylfumarat | O | Vumerity® / Diroximelfumarat | |  |  |
| O | | Aubagio® / Teriflunomid | O | Ponvory® / Ponesimod | |  |  |
| O | | Nerventra® / Laquinimod | O | Other | |  |  |
| O | | Lemtrada™ / Alemtuzumab | *If other medication, which* | |  |  |  |
| O | | Novantron® / Mitoxantron | *---------------------------------------* | | | | |

| **F2.** | **Are you currently using alternative medicine treatments or medications for MS, either in addition to or instead of conventional MS treatment?**  *Please select only of the following answers.* |
| --- | --- |
|  |  |
| O | Yes |
| O | No |

|  |
| --- |

| 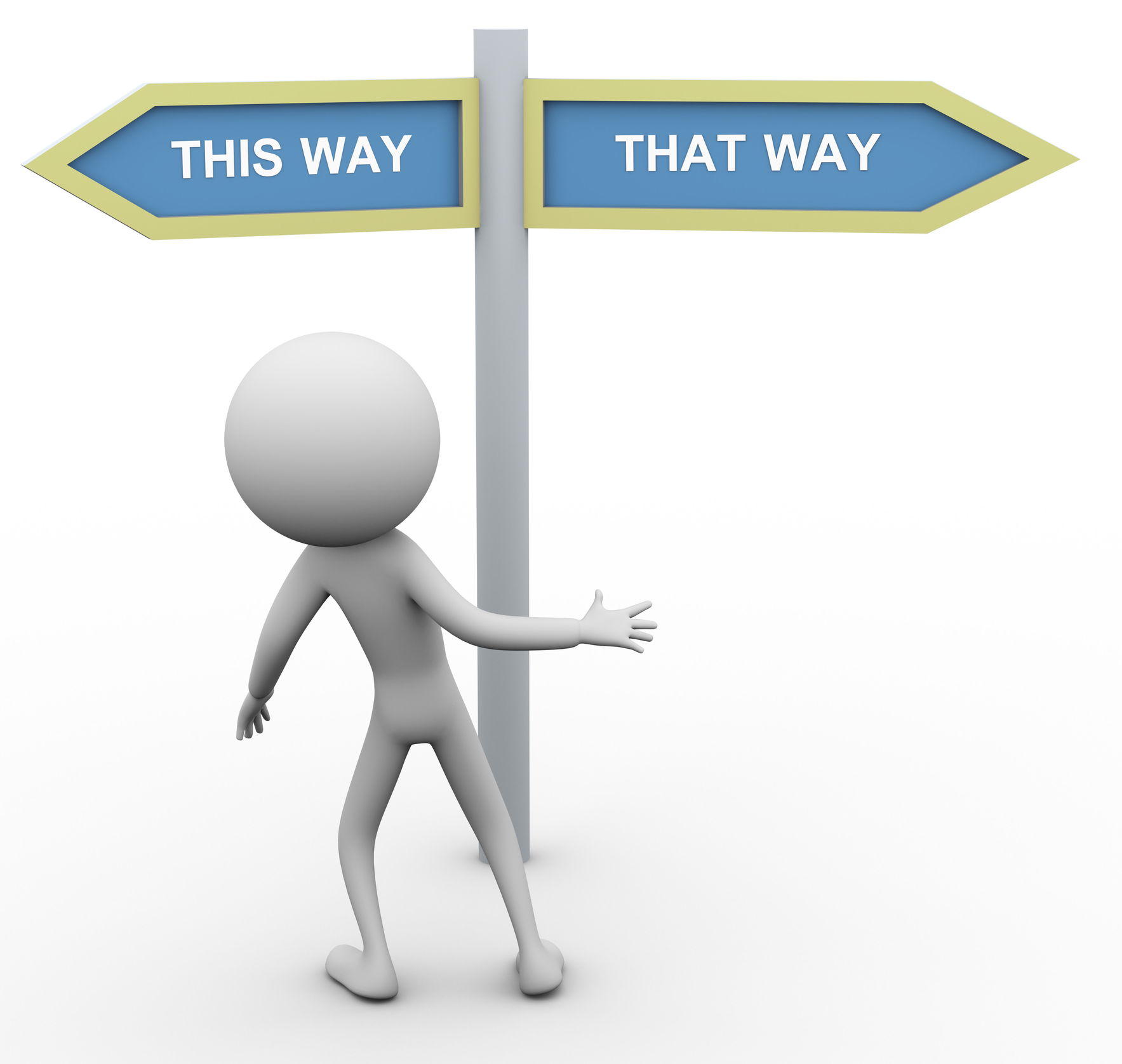 | *If* ***yes*** *to question F2.* *--> continue with question* ***F3*** *(next page).*  *If* ***no*** *--> continue with question* ***F5*** *(page 26).* |
| --- | --- |

|  | |
| --- | --- |
| **F3.** | **Which of the following alternative medical treatments or medications do you take?**  *Please select all applicable answers* |
|  |  |

| O | None | O | Homeopathy |
| --- | --- | --- | --- |
| O | Naturopathy | O | Aromatherapy |
| O | Osteopathy | O | Vitamin B supplements |
| O | TCM | O | Cranberry juice |
| O | Acupuncture | O | Other |
| O | Relaxation therapies |  | *If other alternative medical treatments or medications, which ones?* |
|  |  |  |  |
|  |  |  | *---------------------------------------* |

|  |
| --- |

| **F4.** | **Please state the reason(s) why you are currently undergoing alternative medical treatment.**  *Please select all applicable answers.* |
| --- | --- |
|  |  |
| O | To slow down the progression of the disease. |
| O | To alleviate the symptoms. |
| O | To better cope with the side effects. |
| O | For my own wellbeing. |
| O | Other |
|  | *If other reasons, which ones?* |
|  | *------------------------------------------------* |

|  |
| --- |

| **F5.** | **Are you currently taking any cannabis products for MS?**  *Please select only one of the following answers.* |
| --- | --- |
|  |  |
| O | Yes, after consulting a doctor |
| O | Yes, on my own initiative |
| O | No |

*Comments:*

|  |  |
| --- | --- |

|  |
| --- |

| 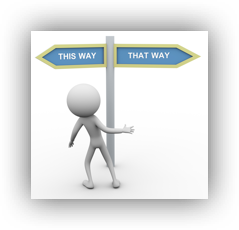 | *If* ***yes*** *--> continue with question* ***F6*** *(next page).*  *If* ***no*** *--> continue with question* ***F9*** *(page 30).* |
| --- | --- |

| **F6.** | Which type of cannabis product do you take? *Please select all applicable answers.* |
| --- | --- |
|  |  |
| O | Sativex |
| O | Cannabis oil |
| O | Cannabis tincture |
| O | Oily mixture |
| O | Street cannabis |
| O | Home-grown |
| O | Only cannabidiol (CBD) |
| O | Other cannabis products |
|  | *If other cannabis products, which ones?* |
|  | *--------------------------------------------* |
|  | |

| **F7.** | **Are these medically prescribed products?**  *Please select only one of the following answers.* |
| --- | --- |
|  |  |
| O | Yes |
| O | No |

*Comments:*

|  |
| --- |

| 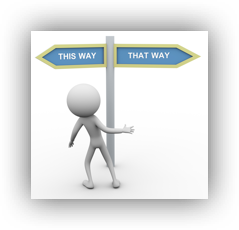 | *If* ***yes*** *--> continue with question* ***F8*** *(next page).*  *If* ***no*** *--> continue with question* ***F9*** *(page 30).* |
| --- | --- |

| **F8.** | **Who pays for the medically prescribed cannabis products?**  *Please write your answer here.* |
| --- | --- |
|  | |

| **F9.** | What non-drug therapies are you currently receiving for MS? *Please select all applicable answers.* |
| --- | --- |
|  |  |
| O | None |
| O | Occupational therapy |
| O | Speech therapy |
| O | Neuropsychological therapy |
| O | Physiotherapy |
| O | Psychotherapy |
| O | Acupuncture |
| O | Osteopathy |
| O | Hippo therapy |
| O | Other |
|  | *If other therapy, which one?* |
|  | *--------------------------------------------* |

|  |
| --- |

**Part G: Side effects or consequences of therapy**

*Below are some questions about unwanted side effects or consequences of therapy.*

| **G1.** | **Have you ever experienced any unwanted side effects or consequences in connection with immunomodulatory MS medication?** | |
| --- | --- | --- |
|  |  | |
| O | Yes | |
| O | No | |
|  | |  |

| 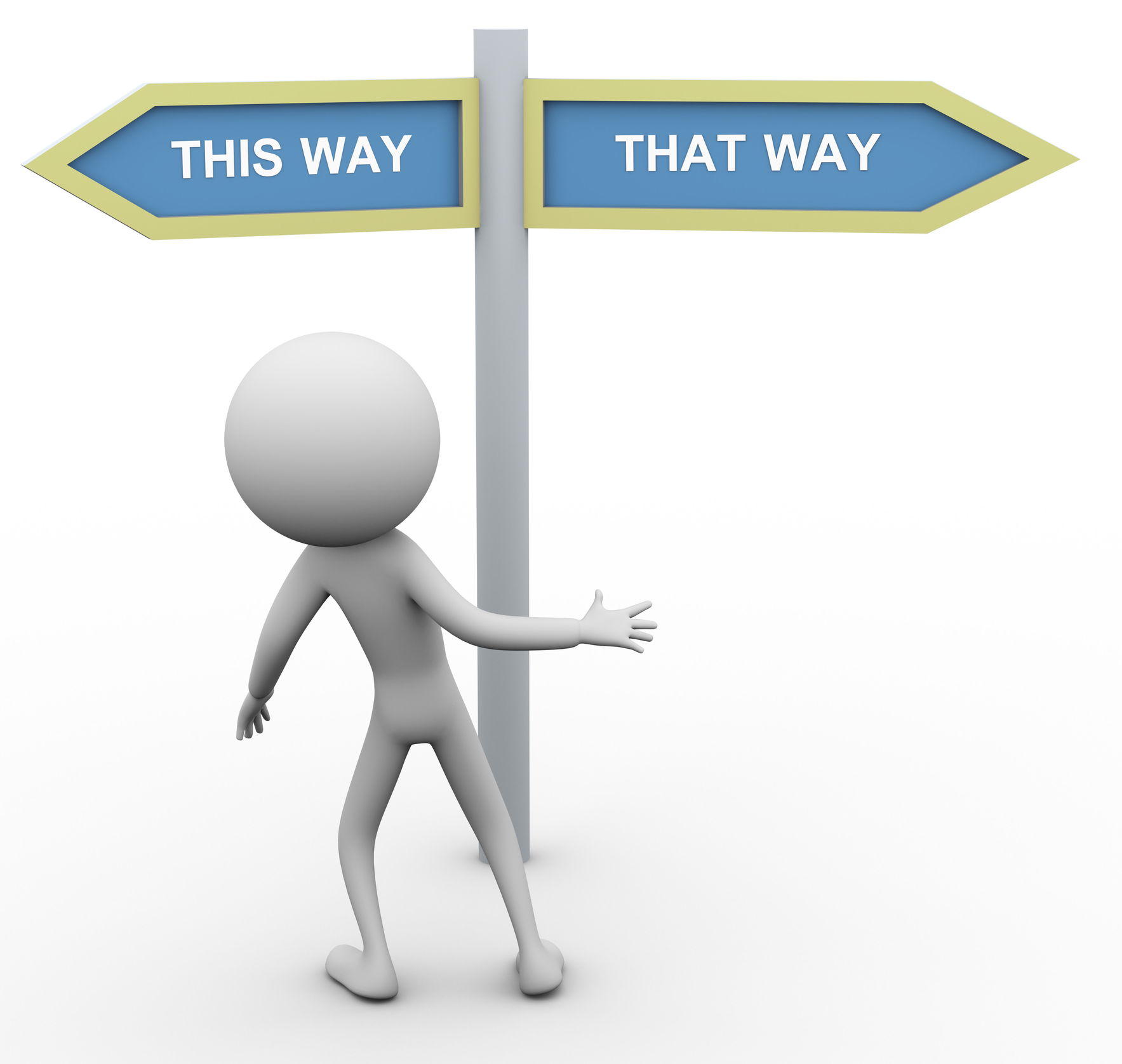 | *If yes --> continue with question* ***G2*** *(next page).*  *If* ***no*** *--> continue with question* ***H1*** *(page 37).* |
| --- | --- |

| **G2.** | | **What was the last immunomodulatory therapy that caused unwanted side effects or consequences?**  *Please select the appropriate answer.* | | |
| --- | --- | --- | --- | --- |
|  | | |  |  |
| \| O \| None \| O \| Imurek® / Azathioprin \| \| \| --- \| --- \| --- \| --- \| --- \| \| O \| Betaferon® / Interferon beta 1b \| O \| Synacthen® / Corticotropin \| \| \| O \| Extavia® / Interferon beta 1b \| O \| Sandimmun® / Cyclosporin \| \| \| O \| Avonex® / Interferon beta 1a \| O \| Endoxan® / Cyclophosphamid \| \| \| O \| Rebif® / Interferon beta 1a \| O \| MabThera® / Rituxan® / Rituximab \| \| \| O \| Plegridy® / Peginterferon beta 1a \| O \| Ocrevus® / Ocrelizumab \| \| \| O \| Copaxone® / Glatirameracetat \| O \| Mavenclad® / Cladribin \| \| \| O \| Gilenya® / Fingolimod \| O \| Mayzent® / Siponimod \| \| \| O \| Tysabri® / Natalizumab \| O \| Arzerra® / Kesimpta® / Ofatumumab \| \| \| O \| Tecfidera® / BG-12 / Dimethylfumarat \| O \| Vumerity® / Diroximelfumarat \| \| \| O \| Aubagio® / Teriflunomid \| O \| Ponvory® / Ponesimod \| \| \| O \| Nerventra® / Laquinimod \| O \| Other \| \| \| O \| Lemtrada™ / Alemtuzumab \| *If other medication, which one?* \| \| \| O \| Novantron® / Mitoxantron \| *---------------------------------------* \| \| \| \| | | | |  |

| **G3.** | **What were these undesirable side effects or consequences? Please also indicate whether you discontinued or interrupted the therapy because of this, or whether you continued the therapy regardless.**  *Please select one answer for each applicable point..* |
| --- | --- |
|  |  |

|  | |  | | **Therapy discontinued** | |  | | **Therapy Interrupted** | |  | | **Therapy continued** | |  |
| --- | --- | --- | --- | --- | --- | --- | --- | --- | --- | --- | --- | --- | --- | --- |
|  | |  |  | |  | |  | |  | |  | |  |  |
|  | Tiredness, Fatigue |  | O | |  | | O | |  | | O | |  | |
|  | Pain |  | O | |  | | O | |  | | O | |  | |
|  | Flu symptoms (e.g. chills, fever) |  | O | |  | | O | |  | | O | |  | |
|  | Sleep problems, Insomnia |  | O | |  | | O | |  | | O | |  | |
|  | Intestinal dysfunction, digestive problems |  | O | |  | | O | |  | | O | |  | |
|  | Dizziness |  | O | |  | | O | |  | | O | |  | |
|  | Hot flashes |  | O | |  | | O | |  | | O | |  | |
|  | Headaches |  | O | |  | | O | |  | | O | |  | |
|  | Allergic reaction |  | O | |  | | O | |  | | O | |  | |
|  | Hair loss |  | O | |  | | O | |  | | O | |  | |
|  | Euphoria |  | O | |  | | O | |  | | O | |  | |
|  | Continuation of symptoms on the next page... |  |  | |  | |  | |  | |  | |  | |
|  | Continuation of symptoms… |  |  | |  | |  | |  | |  | |  | |
|  | Skin problems |  | O | |  | | O | |  | | O | |  | |
|  | Manic or psychotic symptoms |  | O | |  | | O | |  | | O | |  | |
|  | Depression |  | O | |  | | O | |  | | O | |  | |
|  | Measures to prevent PML (progressive multifocal leukoencephalopathy; severe viral infection of the brain), e.g., due to elevated viral titers |  | O | |  | | O | |  | | O | |  | |
|  | Medically diagnosed PML (severe viral infection of the brain, often with a very severe course) |  | O | |  | | O | |  | | O | |  | |
|  |  |  |  | |  | |  | |  | |  | |  | |
|  | Opportunistic infections (infections that typically occur in people with weakened immune systems, e.g., herpes viruses, chronic pneumonia, thrush) |  | O | |  | | O | |  | | O | |  | |
|  | Other |  | O | |  | | O | |  | | O | |  | |
|  | *If other side effects, which ones?* |  |  | |  | |  | |  | |  | |  | |
|  | *-------------------------------------------------* |  |  | |  | |  | |  | |  | |  | |
|  |  |  |  | |  | |  | |  | |  | |  | |

| 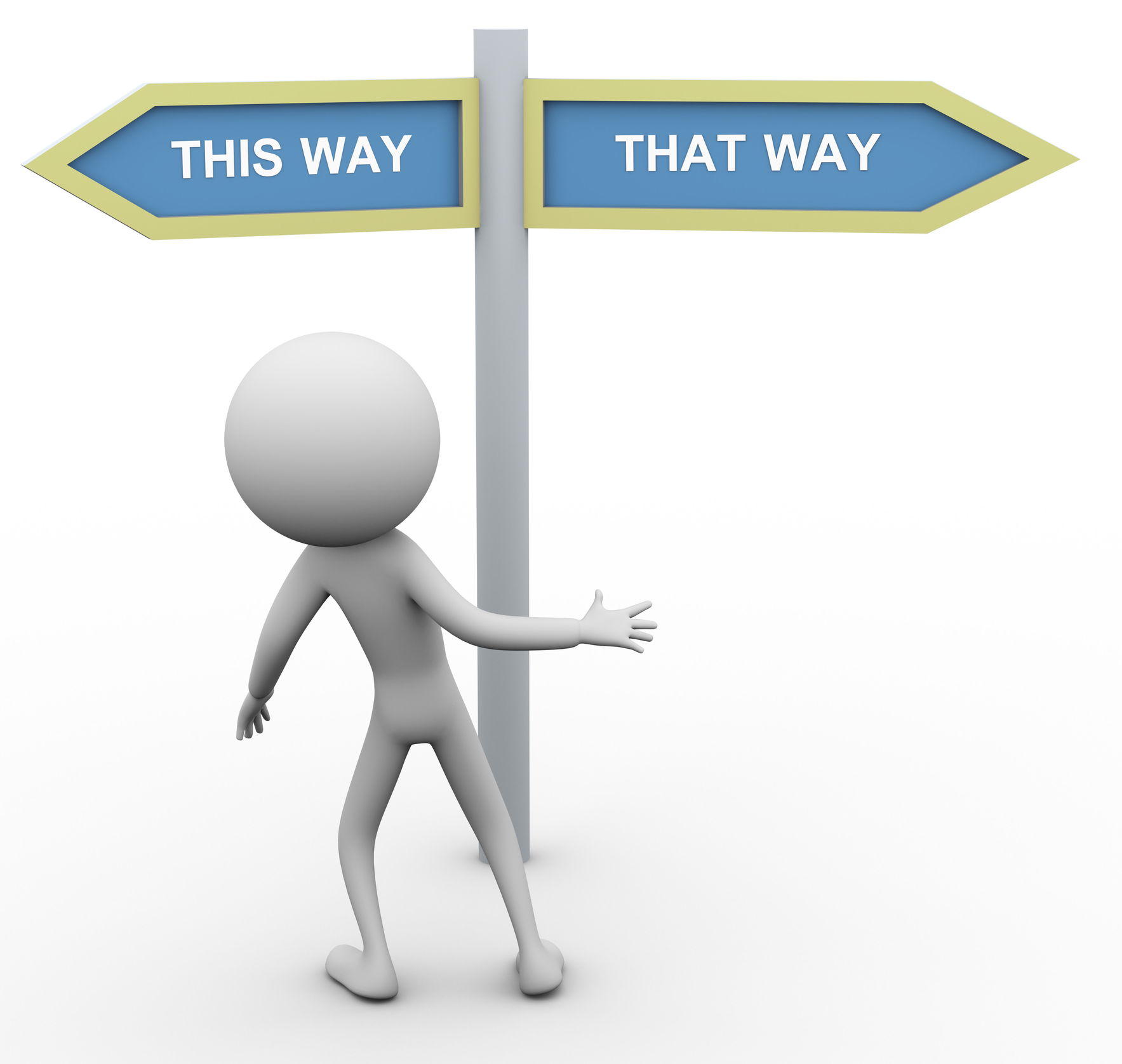 | *If* ***Therapy discontinued / interrupted*** *--> continue with question* ***G4****.*  ***Other*** *--> continue with question* ***H1*** *(page 37).* |
| --- | --- |

| **G4.** | **Was the last interruption or discontinuation of therapy in this regard initiated by a physician? If not, please indicate who initiated it in the comments field.**  *Please select only one of the following answers.* |
| --- | --- |
|  |  |
| O | Yes |
| O | No |
|  | *If not, please explain who initiated it in the comment field.* |
|  |  |

*Comment:*

|  |
| --- |

|  | |
| --- | --- |
| **G5.** | **What further measures were initiated by your doctor?**  *Please select all applicable answers.* |
|  |  |
| O | No further measures |
| O | Change of immunomodulatory therapy suggested/discussed |
| O | Additional medication to treat side effects |
| O | Hospitalization |
| O | Other |
|  | *If other measures, which ones?* |
|  | *----------------------------------------------------* |

**Part H: Other Illnesses**

*Below are some questions about other illnesses. To understand MS better, it is important to know which other illnesses occur together with MS.*

| **H1.** | **Do you have any other diseases besides MS? If so, please specify which ones.**  *Please select all applicable answers.* |
| --- | --- |

| O | No, none | | O | Orthopedic conditions  (e.g. joint or back pain) |  |
| --- | --- | --- | --- | --- | --- |
| O | Diabetes Type I (insulin-dependent, early onset) | | O | Skin conditions (e.g. acne) |  |
| O | Diabetes Type II (formerly known as adult-onset diabetes) | | O | Psoriasis |  |
| O | Cancer | | O | Asthma |  |
| O | High blood pressure | | O | Other |  |
| O | Heart problems | |  | *If other illnesses, which ones?* |  |
| O | Depression | |  |  |  |
| O | Osteoporosis | |  | *-------------------------------------------* |  |
| O | Rheumatic illnesses (e.g. Arthritis, Gout) | |  | *-------------------------------------------* |  |
|  | |  | | | |

|  |
| --- |

| **H2.** | **What medications/supplements do you take in connection with these illnesses?**  *Please note these details in the corresponding fields* |
| --- | --- |

| O | None |
| --- | --- |
| 1. Medication |  |
| 2. Medication |  |
| 3. Medication |  |
| 4. Medication |  |
| 5. Medication |  |
| 6. Medication |  |
| 7. Medication |  |
| 8. Medication |  |
| 9. Medication |  |
| 10. Medication |  |

|  |
| --- |

**Part I: Risk factors and health questions**

*Below are some questions about possible risk factors and health issues.*

| **I1.** | **How tall are you (in cm)?** |
| --- | --- |
| \|  \|  \|  \| Height in cm \| \| --- \| --- \| --- \| --- \| | |

| **I2.** | **How heavy are you (in kg)?** |
| --- | --- |
| \|  \|  \|  \| Weight in kg \| \| --- \| --- \| --- \| --- \| | |

| **I3.** | **Apart from MS or other illnesses you may have noted previously, have you ever had/do you still have any of the following conditions?**  *Please select all applicable answers for each item.* |
| --- | --- |
|  |  |

|  | | |  | | **Yes** |  | | **Unsure** |  | | **No** |  |  |
| --- | --- | --- | --- | --- | --- | --- | --- | --- | --- | --- | --- | --- | --- |
|  | | |  |  | |  |  | |  |  | |  |  |
|  | | Pfeiffer's glandular fever/ “kissing disease”/ Mononucleosis |  | O | |  | O | |  | O | |  | |
|  | | Migraines |  | O | |  | O | |  | O | |  | |
|  | | Angina (throat/tonsil inflammation) |  | O | |  | O | |  | O | |  | |
|  | | Hay fever |  | O | |  | O | |  | O | |  | |
|  | | Food allergies / intolerances |  | O | |  | O | |  | O | |  | |
|  | | Drug allergies |  | O | |  | O | |  | O | |  | |
|  | | Herpes (including „  cold sores“) |  | O | |  | O | |  | O | |  | |
|  | | Eczema / Neurodermatitis |  | O | |  | O | |  | O | |  | |
|  | | Cystitis |  | O | |  | O | |  | O | |  | |
|  | | Rheumatoid arthritis |  | O | |  | O | |  | O | |  | |
|  | | Irritable bowel syndrome |  | O | |  | O | |  | O | |  | |
|  | | Ulcerative colitis (chronic inflammatory disease of the colon) |  | O | |  | O | |  | O | |  | |
|  | |  |  |  | |  |  | |  |  | |  | |
|  | | Crohn’s disease (chronic inflammatory disease of the digestive tract) |  | O | |  | O | |  | O | |  | |
|  | | Ulcer (stomach ulcer) |  | O | |  | O | |  | O | |  | |
|  | | | | | | | | | | | |  |  |

| **I4.** | **Have you had any other illnesses or suffered any injuries, such as traumatic brain injury or concussion? If so, please specify.**  *Please select only one of the following answers.* |
| --- | --- |
|  |  |
| O | Yes |
| O | No |

*Comments:*

|  |
| --- |

| **I5.** | **Have you ever smoked?**  *Please select only one of the following answers.* |
| --- | --- |
|  |  |
| O | Never |
| O | In the past |
| O | I still smoke |
|  |  |

|  |
| --- |

| 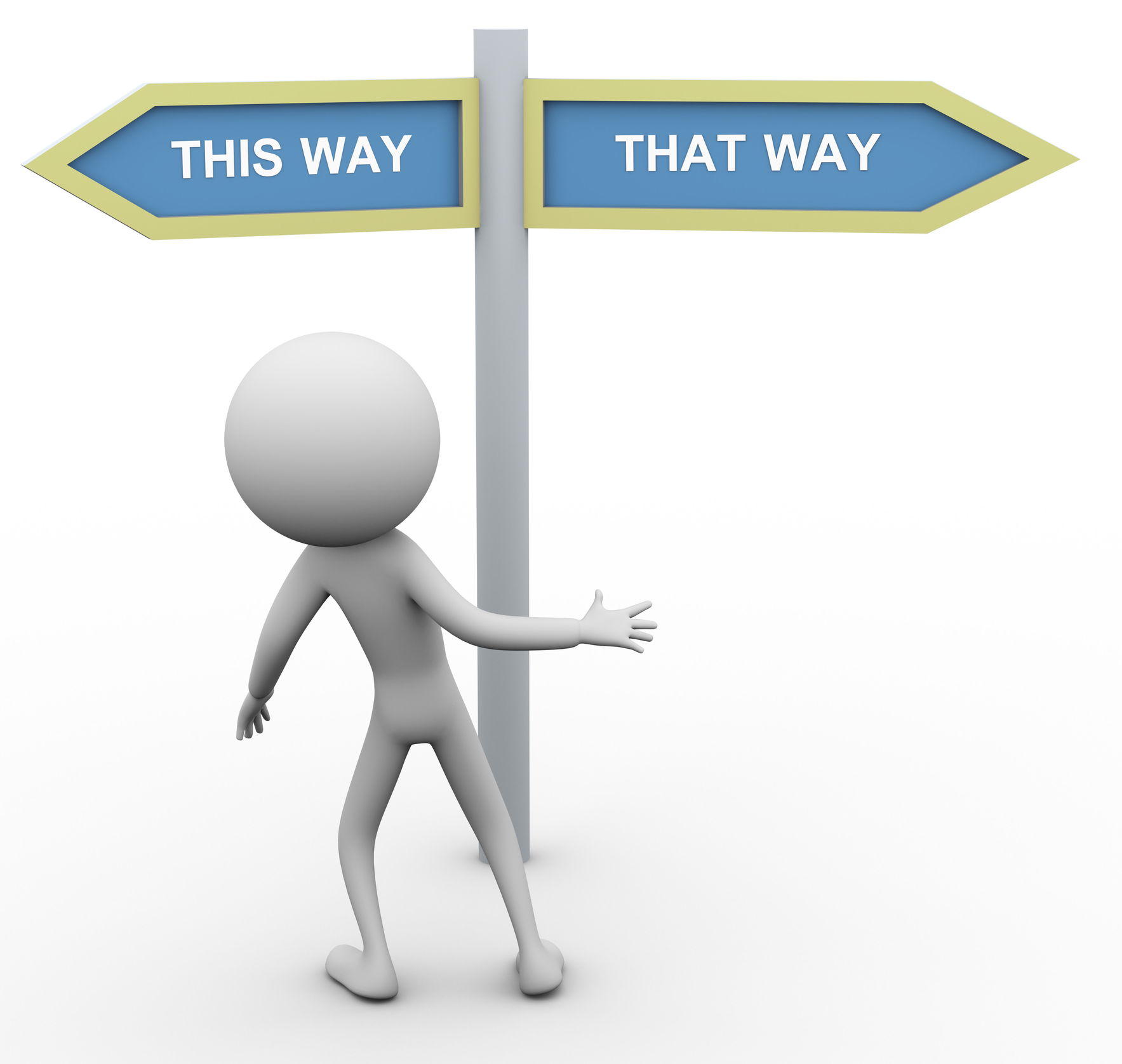 | *If you answered* ***in the past or I still smoke*** *--> continue with question* ***I6****.*  ***Otherwise*** *--> continue with question* ***I9*** *(page 44).* |
| --- | --- |

| **I6.** | **How many cigarettes do you smoke/did you smoke on average per day?**  *Only numbers may be entered in this field.* |
| --- | --- |
| \|  \|  \| Cigarettes per day \| \| --- \| --- \| --- \| | |

|  |
| --- |

| **I7.** | **When did you start smoking? Please specify the year.** |
| --- | --- |
| \|  \|  \|  \|  \| Year \| \| --- \| --- \| --- \| --- \| --- \| \|  \|  \|  \|  \|  \| | |

|  | |
| --- | --- |
| **I8.** | **How long did you smoke?**  *Please select only one answer.* |
|  |  |
| O | Less than 1 year |
| O | 1-5 years |
| O | 5-10 years |
| O | 10-15 years |
| O | 15-20 years |
| O | 20-30 years |
| O | 30-40 years |
| O | More than 40 years |
| O | Other |
|  | *If other time period, which one?* |
|  | *------------------------------------------------* |
| O | I still smoke |

| **I9.** | How often do you consume alcoholic beverages? *Please select only one of the following answers.* |
| --- | --- |
|  |  |
| O | Never |
| O | Seldom |
| O | 1x / Month |
| O | 2-3x / Month |
| O | 1-2x / Week |
| O | 2-3x / Week |
| O | 3-6x / Week |
| O | Daily |

|  |
| --- |

| 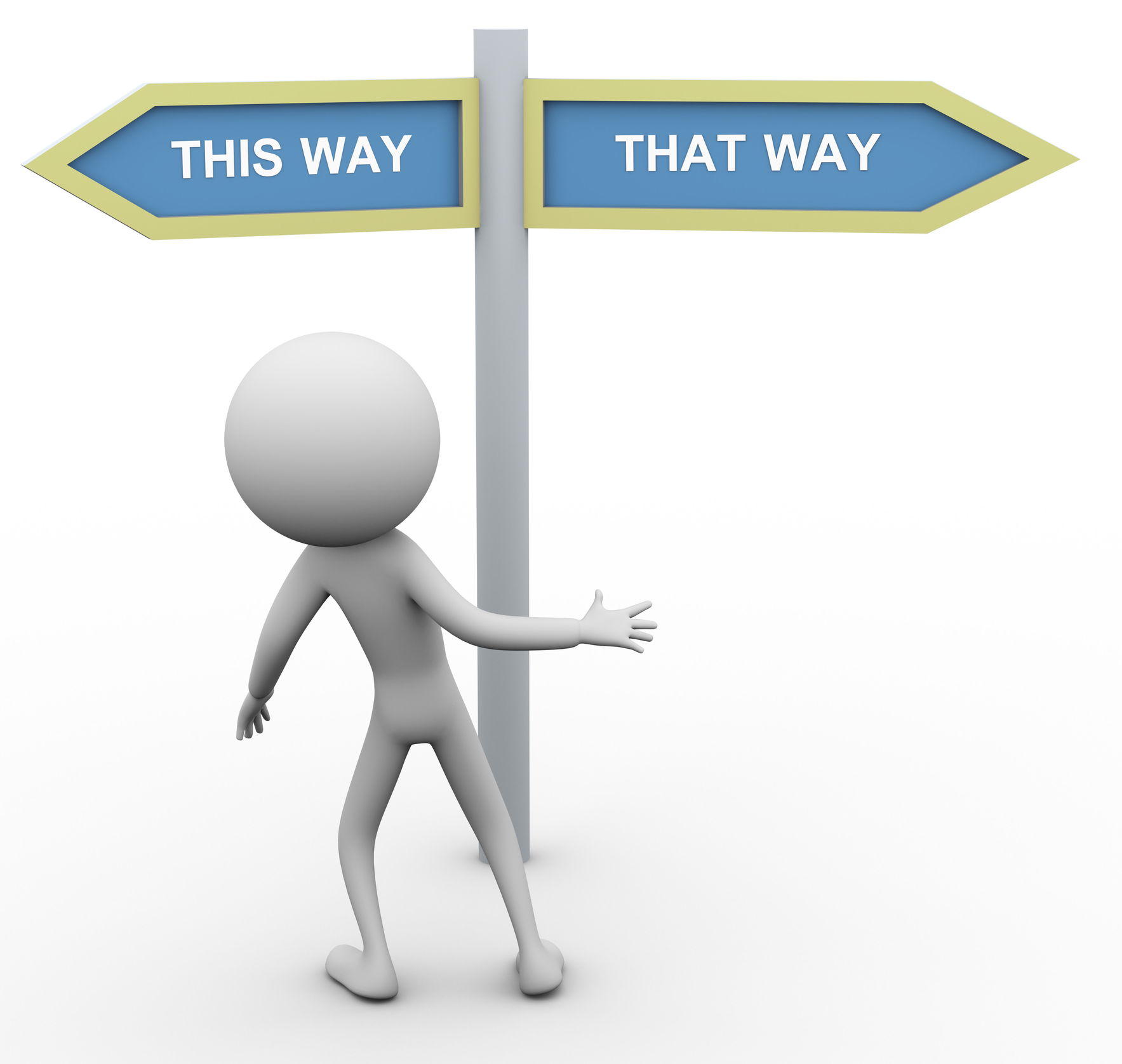 | *If you consume alcoholic beverages* ***seldom or more frequently*** *--> continue with question* ***I10****.*  *If* ***never*** *--> continue with question* ***I11.*** |
| --- | --- |

| **I10.** | **What alcoholic beverages do you consume?**  *Please select all applicable answers.* |
| --- | --- |
|  |  |
| O | Beer |
| O | Wine |
| O | Mixed Drinks / Cocktails |
| O | Spirits |
| O | Other |
|  | *If other beverages, which?* |
|  | *---------------------------------------------* |

|  |
| --- |

| **I11.** | **Did anyone help you fill out the questionnaire (enter the answers)? If so, please indicate whom.**  *Please select only one of the following answers.* |
| --- | --- |
|  |  |
| O | Yes |
| O | No |
|  |  |

*Comments:*

|  |
| --- |

|  | **Do you have any further comments, suggestions or additions?** |
| --- | --- |
|  |  |

|  |  |
| --- | --- |
|  |  |
|  |  |
|  |  |
|  |  |
|  |  |
|  |  |
|  |  |
|  |  |
|  |  |
|  |  |

*Thank you very much! This was the last question of the main survey.*

*We will contact you again in a few months to invite you to take part in another follow-up survey.*

*Until then, we wish you all the best!*

*Your MS Register Team*

Please return the completed questionnaire with the enclosed prepaid envelope to the MS Registry Center.

We are available to answer any questions you may have during office hours:

Tel. 044 634 48 59

Email: ms-register@ebpi.uzh.ch

ö

**Additional information about your**

**personal situation**

**MS REGISTRY**


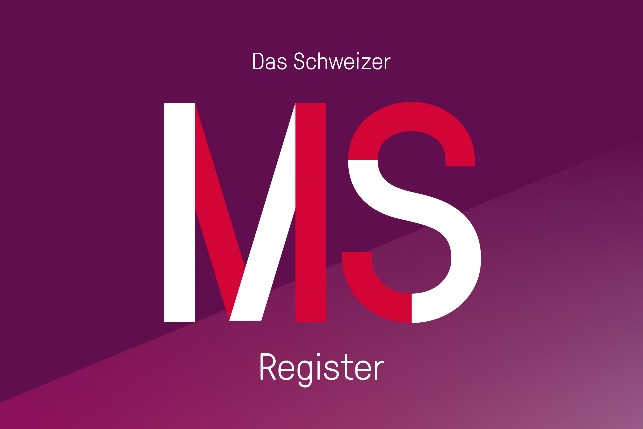


**Swiss MS Registry**

**2023**

**Part A: Personal Data**

*Below you will find some questions about yourself.*

| **A1.** | **Please enter your year of birth.**  *Only numbers may be entered in this field.*   \| *E.g.* \| 1 \| 9 \| 6 \| 7 \| Year \|  \| \| --- \| --- \| --- \| --- \| --- \| --- \| --- \| |
| --- | --- | --- | --- | --- | --- | --- | --- | --- |
| \|  \|  \|  \|  \|  \| Year \|  \| \| --- \| --- \| --- \| --- \| --- \| --- \| --- \| | |

| **A2.** | **Please state your place of birth below.** |
| --- | --- |
|  | |

| **A3.** | **If you were not born in Switzerland, in which year did you come to Switzerland?**  *Only numbers may be entered in this field.* |
| --- | --- |
| \|  \|  \|  \|  \|  \| Year \|  \| \| --- \| --- \| --- \| --- \| --- \| --- \| --- \| | |

| **A4.** | **What is your nationality?**  *Multiple answers possible.* |
| --- | --- |

| O | Swiss | O | Croatian |
| --- | --- | --- | --- |
| O | Albanian | O | Macedonian |
| O | Bosnian | O | Portuguese |
| O | German | O | Serb |
| O | French | O | Spanish |
| O | Greek | O | Turk |
| O | Italian | O | Another country |
| O | Kosovars |  | *If another country, which one?*  *-----------------------------------------* |
|  |  |  | *-----------------------------------------* |
|  |  |  | *-----------------------------------------* |

| **A5.** | **What is your connection to the Swiss MS Society?**  *Multiple answers possible.* |
| --- | --- |
|  |  |
| O | I am a member of the Swiss MS Society. |
| O | I am a donor or patron |
| O | I regularly or sporadically obtain information from the Swiss MS Society (e.g. newsletter, brochures, homepage, Facebook friend). |
| O | I participate in a regional group of the Swiss MS Society. |
| O | I volunteer my time to help with activities of the Swiss MS Society. |
| O | I have no connection to the Swiss MS Society. |
| O | Other |
|  | *In case of other connections, which?* |
|  | *----------------------------------------------* |

|  | |
| --- | --- |
|  |  |

**Part B: Disease progression**

*The following questions relate to the progression of MS (for confirmed and suspected MS diagnoses) and the occurrence of MS within your family or amongst relatives.*

|  | |
| --- | --- |
| **B1.** | **Do you have a medically confirmed diagnosis of MS?**  *Please select only one of the following answers.* |
|  |  |
| O | Yes, confirmed diagnosis. |
| O | No, presumed diagnosis. |
|  | *Any further comments?* |

|  |
| --- |

| **B2.** | **Do any family members or relatives (blood relatives only) have a confirmed diagnosis of MS?**  *Please select only one of the following answers.* |
| --- | --- |
|  |  |
| O | Yes |
|  | *If yes, degree of kinship (e.g. mother, maternal grandfather)?* |

| O | No |
| --- | --- |
| O | I don’t know |

|  | *Additional comments:* |
| --- | --- |

*The following question refers to you again.*

| **B3.** | **Please indicate the current stage of your MS disease.**  *Please select only one of the following answers.* |
| --- | --- |
|  |  |
| O | Clinically isolated syndrome (CIS) |
| O | Relapsing remitting MS (RRMS) |
| O | Primary progressive MS (PPMS) |
| O | Secondary progressive MS (SPMS) |
| O | Transition between two stages or other forms |
|  | *Any further comments?* |

|  |
| --- |

| 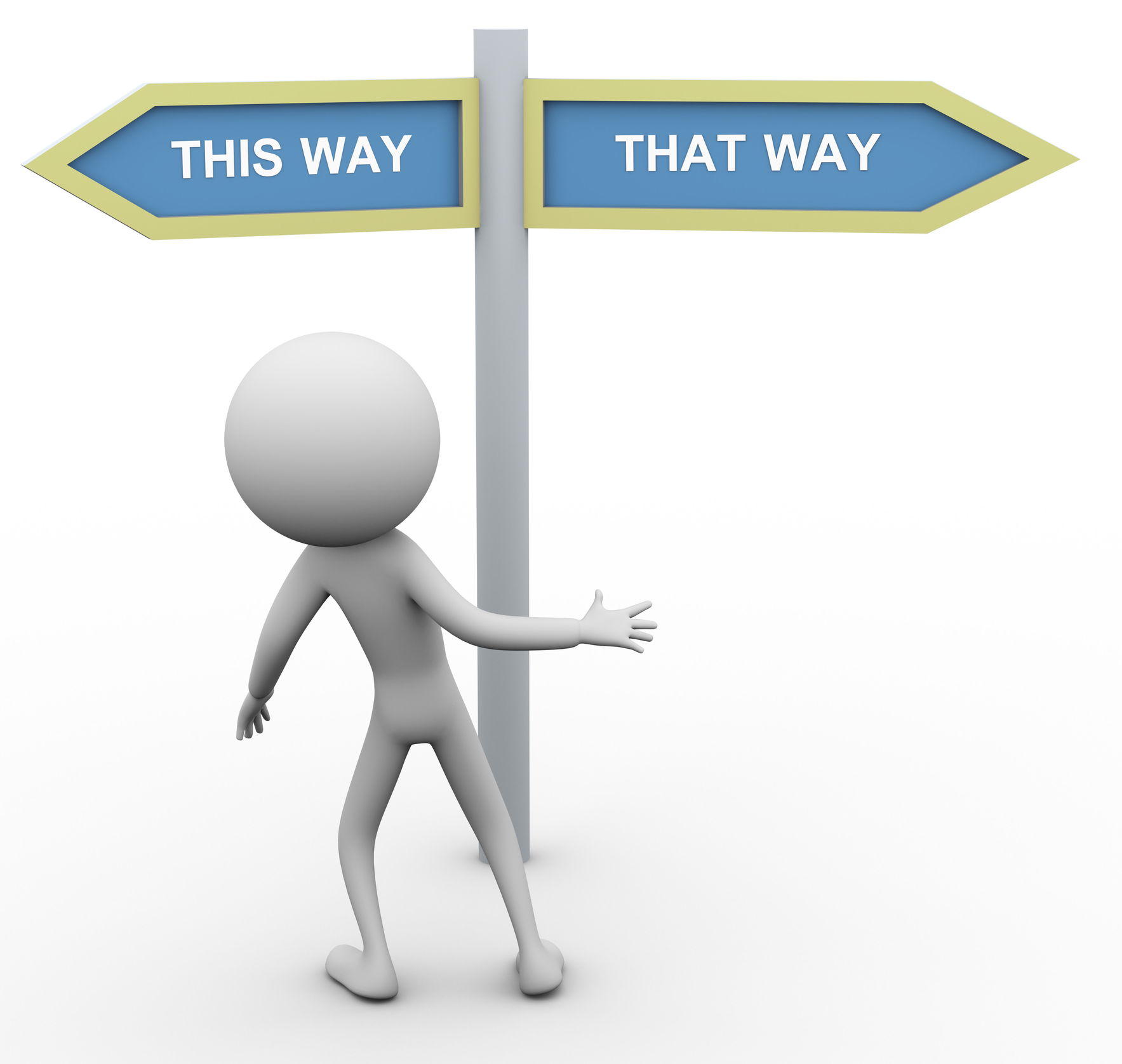 | *If you answered* ***yes, confirmed MS diagnose for question B1***  *--> continue with question* ***B4****.*  ***Otherwise*** *--> continue with question* ***B5*** |
| --- | --- |

| **B4.** | **When were you diagnosed with MS? Please indicate the month (if known) and the year.**  *Only numbers may be entered in this field.*   \| *E.g.* \| 0 \| 7 \| Month \| 2 \| 0 \| 0 \| 4 \| Year \|  \| \| --- \| --- \| --- \| --- \| --- \| --- \| --- \| --- \| --- \| --- \| |
| --- | --- | --- | --- | --- | --- | --- | --- | --- | --- | --- | --- |
| \|  \|  \|  \| Month \|  \|  \|  \|  \| Year \|  \| \| --- \| --- \| --- \| --- \| --- \| --- \| --- \| --- \| --- \| --- \| | |

| **B5.** | **When did the first symptoms appear (also prior to diagnosis)? Please specify the month (if known) and the year.**  *Only numbers may be entered in this field.*   \| *E.g.* \| 1 \| 2 \| Month \| 1 \| 9 \| 9 \| 9 \| Year \| \| --- \| --- \| --- \| --- \| --- \| --- \| --- \| --- \| --- \| |
| --- | --- | --- | --- | --- | --- | --- | --- | --- | --- | --- |
| \|  \|  \|  \| Month \|  \|  \|  \|  \| Year \|  \| \| --- \| --- \| --- \| --- \| --- \| --- \| --- \| --- \| --- \| --- \| | |

| **B6.** | **Which of the following symptoms have you experienced in connection with MS? Please indicate which of these initial symptoms were and which appeared later.**  *Please select only one of the following answers for each applicable symptom.* |
| --- | --- |
|  |  |

|  | |  | |  | **Initial symptom** | **Appeared later** |  | |
| --- | --- | --- | --- | --- | --- | --- | --- | --- |
|  |  |  |  | |  |  | |  |
|  | Visual disturbances |  |  | | O | O | |  |
|  | Speech disorders |  |  | | O | O | |  |
|  | Swallowing disorders (Dysphagia) |  |  | | O | O | |  |
|  | Weakness |  |  | | O | O | |  |
|  | Paralysis |  |  | | O | O | |  |
|  | Tiredness, fatigue |  |  | | O | O | |  |
|  | Sensory disturbances (e.g. numbness, tingling) |  |  | | O | O | |  |
|  | Dizziness |  |  | | O | O | |  |
|  | Pain |  |  | | O | O | |  |
|  | Gait disorder |  |  | | O | O | |  |
|  | *Continuation of symptoms on the next page…* |  |  | |  |  | |  |
|  | *Continuation of symptoms...* |  |  | |  |  | |  |
|  | Balance disorders |  |  | | O | O | |  |
|  | Bladder disorders (e.g. weak bladder) |  |  | | O | O | |  |
|  | Spasms (muscle cramps) |  |  | | O | O | |  |
|  | Twitching, tics |  |  | | O | O | |  |
|  | Tremor (shaking) |  |  | | O | O | |  |
|  | Bowel disorders (e.g. constipation) |  |  | | O | O | |  |
|  | Epileptic seizures |  |  | | O | O | |  |
|  | Sexual dysfunction |  |  | | O | O | |  |
|  | Memory impairment |  |  | | O | O | |  |
|  | Depression |  |  | | O | O | |  |
|  | Other symptoms |  |  | | O | O | |  |
|  | *If others, please specify:* |  |  | | O | O | |  |
|  | ------------------------------ |  |  | |  |  | |  |
|  | *If others, please specify* |  |  | | O | O | |  |
|  | ------------------------------ |  |  | |  |  | |  |

**Part C: Therapies**

*The following section concerns any therapies or treatments you have already received*.

| **C1.** | **Have you ever received MS-specific drug treatment?**  *Please select only one of the following answers.* |
| --- | --- |
|  |  |
| O | Yes |
| O | No |

|  |
| --- |

| 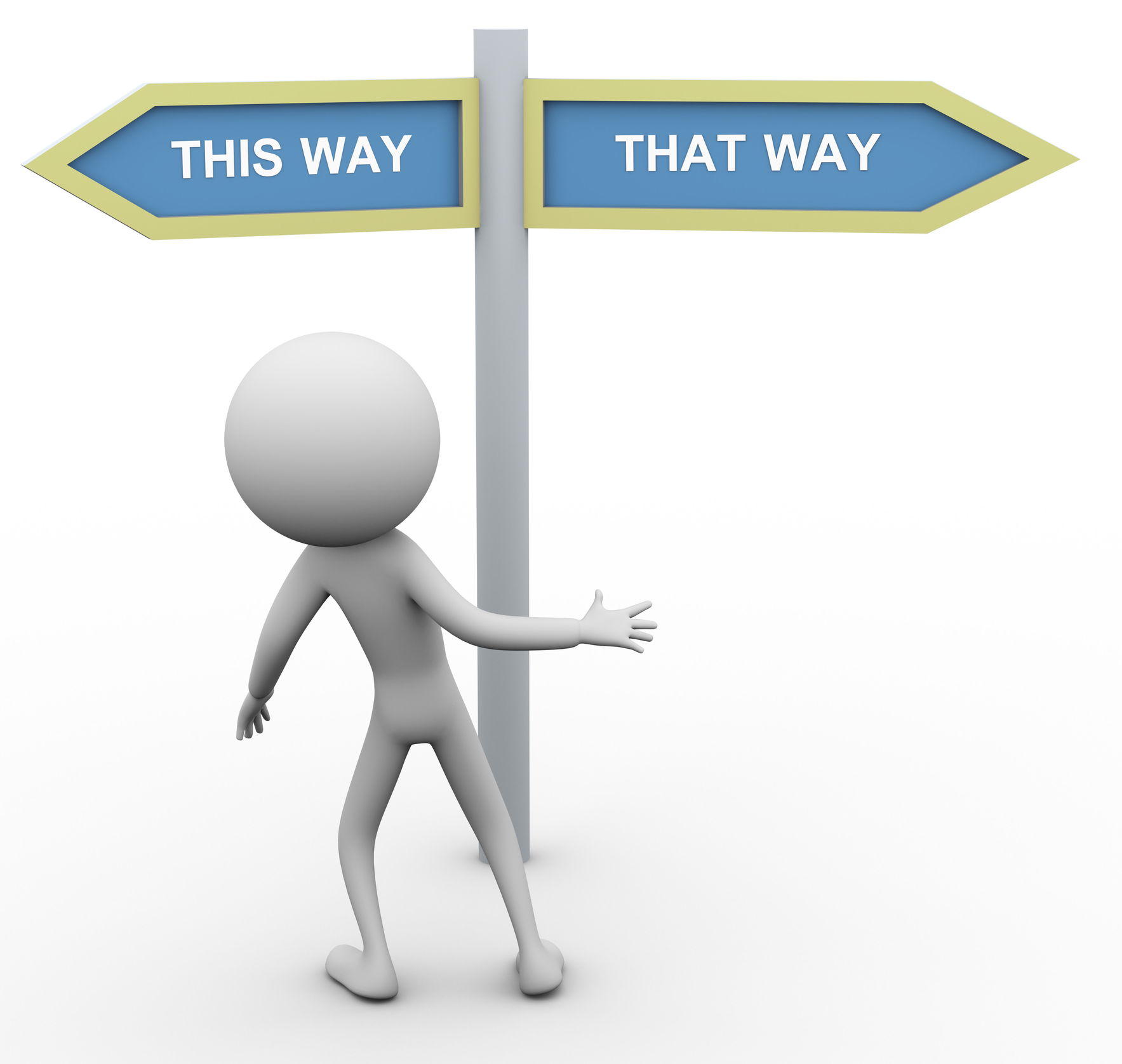 | *If* ***yes*** *--> continue with question* ***C2****.*  *If* ***no*** *--> continue with question* ***C4*** *(page 18).* |
| --- | --- |

| **C2.** | **When did you receive your first MS treatment? Please indicate the month (if known) and the year.**  *Only numbers may be entered in this field.*   \| *E.g.* \| 0 \| 5 \| Month \| 2 \| 0 \| 0 \| 1 \| Year \|  \| \| --- \| --- \| --- \| --- \| --- \| --- \| --- \| --- \| --- \| --- \| |
| --- | --- | --- | --- | --- | --- | --- | --- | --- | --- | --- | --- |
| \|  \|  \|  \| Month \|  \|  \|  \|  \| Year \|  \| \| --- \| --- \| --- \| --- \| --- \| --- \| --- \| --- \| --- \| --- \| | |

| **C3.** | **Have you previously received any of the following treatments for MS?**  *Please select the appropriate answer for the corresponding medications (tick the relevant time period).* |
| --- | --- |
|  |  |

|  | |  | | **More than 6 months ago** | |  | | **Within the last 6 months** | |  | |  | |  |  |
| --- | --- | --- | --- | --- | --- | --- | --- | --- | --- | --- | --- | --- | --- | --- | --- |
|  | |  |  | |  | |  | |  | |  | |  | |  |
|  | |  |  | |  | |  | |  | |  | |  | |  |
|  | Betaferon® / Interferon beta 1b |  | O | |  | | O | |  | |  | |  | |  |
|  | Extavia® / Interferon beta 1b |  | O | |  | | O | |  | |  | |  | |  |
|  | Avonex® / Interferon beta 1a |  | O | |  | | O | |  | |  | |  | |  |
|  | Rebif® / Interferon beta 1a |  | O | |  | | O | |  | |  | |  | |  |
|  | Plegridy® / Peginterferon beta 1a |  | O | |  | | O | |  | |  | |  | |  |
|  | Copaxone® / Glatirameracetat |  | O | |  | | O | |  | |  | |  | |  |
|  | Gilenya® / Fingolimod |  | O | |  | | O | |  | |  | |  | |  |
|  | Tysabri® / Natalizumab |  | O | |  | | O | |  | |  | |  | |  |
|  | Tecfidera® / BG-12 / Dimethylfumarat |  | O | |  | | O | |  | |  | |  | |  |
|  | Aubagio® / Teriflunomid |  | O | |  | | O | |  | |  | |  | |  |
|  | *Continuation of medication on the next page...* |  |  | |  | |  | |  | |  | |  | |  |
|  | *Continuation of the medication...* |  |  | |  | |  | |  | |  | |  | |  |
|  | Nerventra® / Laquinimod |  | O | |  | | O | |  | |  | |  | |  |
|  | Lemtrada™ / Alemtuzumab |  | O | |  | | O | |  | |  | |  | |  |
|  | Novantron® / Mitoxantron |  | O | |  | | O | |  | |  | |  | |  |
|  | Imurek® / Azathioprin |  | O | |  | | O | |  | |  | |  | |  |
|  | Synacthen® / Corticotropin |  | O | |  | | O | |  | |  | |  | |  |
|  | Sandimmun® / Cyclosporin |  | O | |  | | O | |  | |  | |  | |  |
|  | Endoxan® / Cyclophosphamid |  | O | |  | | O | |  | |  | |  | |  |
|  | MabThera® / Rituximab |  | O | |  | | O | |  | |  | |  | |  |
|  | Ocrevus® / Ocrelizumab |  | O | |  | | O | |  | |  | |  | |  |
|  | Mavenclad® / Cladribin |  | O | |  | | O | |  | |  | |  | |  |
|  | Cortisone |  | O | |  | | O | |  | |  | |  | |  |
|  | Others |  | O | |  | | O | |  | |  | |  | |  |
|  |  |  |  | |  | |  | |  | |  | |  | |  |

| **C4.** | **Have you ever received treatment using alternative medicine methods as a complement to or instead of conventional treatment for MS?**  *Please select only one of the following answers.* |
| --- | --- |
|  |  |
| O | Yes |
| O | No |
|  |  |

*Additional information on the nature of these methods will be collected in a later survey.*

| **C5.** | **Are you currently taking any additional medication (e.g. conventional painkillers such as Dafalgan® / Paracetamol etc.)?**  *Please select only one of the following answers.* |
| --- | --- |
|  |  |
| O | Yes |
| O | No |
|  |  |

*Additional information on the type of these medications will be collected in a later survey.*

**Part D: Lifestyle factors and final questions**

*The following question relates to lifestyle factors and whether these have changed because of MS.*

| **D1.** | **Have you made any changes in the following areas of your life because of MS?**  *Please select the appropriate answer for each item. If any of the items do not apply to you, please skip them.* |
| --- | --- |

|  | |  | | **Less** | |  | | **Same** |  | | | **More** |  |
| --- | --- | --- | --- | --- | --- | --- | --- | --- | --- | --- | --- | --- | --- |
|  | |  |  | |  | |  | | |  |  | |  |
|  | Physical activity / sport |  | O | |  | | O | | |  | O | |  |
|  | Conscious approach to stress |  | O | |  | | O | | |  | O | |  |
|  | Smoking |  | O | |  | | O | | |  | O | |  |
|  | Alcohol consumption |  | O | |  | | O | | |  | O | |  |
|  | Regular sleep-wake cycle |  | O | |  | | O | | |  | O | |  |
|  | Methods of relaxation (e.g. autogenic training, walking, etc.) |  | O | |  | | O | | |  | O | |  |
|  | Social contact / activities |  | O | |  | | O | | |  | O | |  |
|  | Work-life balance |  | O | |  | | O | | |  | O | |  |
|  | Healthy diet |  | O | |  | | O | | |  | O | |  |
|  | Other |  | O | |  | | O | | |  | O | |  |
|  | *If other areas of life, please specify:* |  |  | |  | |  | | |  |  | |  |
|  | *--------------------------------------------------* |  |  | |  | |  | | |  |  | |  |

*These and other areas of life are addressed in a separate survey entitled “My Life with MS”.*

*Finally, there are a few questions about the next steps and future participation in the MS Registry.*

*The next question is especially crucial for confirming participation in the MS Registry’s ongoing surveys.*

| **D2.** | **Would you be willing to participate in the semi-annual surveys of the Swiss MS Registry? If yes, you will receive further documents from us in about 4 weeks.**  *Please select only one of the following answers.* |
| --- | --- |
|  |  |
| O | Yes |
| O | No |

|  | *Any additional comments?* |
| --- | --- |

|  |
| --- |

| **D3.** | | **May we send you information about additional, voluntary studies conducted outside of the Swiss MS Registry in the future?**  **These selected studies will have been previously assessed as meaningful and worthy of support by the Research Commission of the MS Registry.**  **IMPORTANT: Your contact details will not be shared without your consent.**  *Please select only one of the following answers.* |  |
| --- | --- | --- | --- |
|  | |  |  |
| O | Yes | | |
| O | No | | |
|  | *Any additional comments?* | | |

|  |
| --- |

| **D4a.** | **Which doctor is mainly responsible for the treatment of your MS? Please provide the name of the doctor as well as the address of the practice or clinic in the comment field.**  *Please write your answer here.* |
| --- | --- |
|  | |

| **D4b.** | **Which practice, clinic, or rehabilitation clinic have you also visited in the past year? Please provide the corresponding address in the comment field.**  *Please enter your answer here.* |
| --- | --- |
|  | |

|  | **Would you like to add any further comments, suggestions or additions?** |
| --- | --- |
|  |  |

|  |  |
| --- | --- |
|  |  |
|  |  |
|  |  |
|  |  |
|  |  |
|  |  |
|  |  |
|  |  |
|  |  |
|  |  |

***This was the final question regarding additional personal information.*** *We greatly appreciate your participation in the Swiss MS Registry and would like to sincerely thank you for your contribution!*

*If you would like to take part in the ongoing surveys of the MS Registry (Question D2 on page 21), you will receive further documents from us shortly.*

*With kind regards*

*Your MS Registry Team*

Please return the completed questionnaire to the MS Registry Center using the enclosed prepaid envelope.

If you have any questions, we are happy to assist you during office hours:

Tel. 044 634 48 59

Email: ms-register@ebpi.uzh.ch

| **H6.** | **Did the initial diagnosis consultation lead to you starting immunomodulatory therapy within a certain period of time afterwards?**  *Please select only one of the following answers.* |
| --- | --- |
|  |  |
| O | Yes, therapy started within 2 weeks |
| O | Yes, therapy started within 1 month |
| O | Yes, therapy started within 3 months |
| O | Yes, but therapy started after more than 3 months |
| O | No |
| O | Other |
|  | *If other, please comment here:* |

| 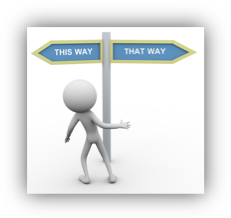 | *If* ***Yes*** *or* ***Other*** *--> continue with question* ***H7****.*  *If* ***No***  *--> continue with question* ***H10****.* |
| --- | --- |

| **H7.** | **Which immunomodulatory therapy did you start with based on the initial diagnosis consultation?**  *Please select only one of the following answers.* |
| --- | --- |
|  |  |

| O | Betaferon® / Interferon beta 1b |
| --- | --- |
| O | Extavia® / Interferon beta 1b |
| O | Avonex® / Interferon beta 1a |
| O | Rebif® / Interferon beta 1a |
| O | Plegridy® / Peginterferon beta 1a |
| O | Copaxone® / Glatirameracetat |
| O | Gilenya® / Fingolimod |
| O | Tysabri® / Natalizumab |
| O | Tecfidera® / BG-12 |
| O | Aubagio® / Teriflunomid |
|  | *Continuation of the medication on the next page...* |

|  | *Continuation of medication...* |
| --- | --- |
| O | Nerventra® / Laquinimod |
| O | Lemtrada™ / Alemtuzumab |
| O | Novantron® / Mitoxantron |
| O | Imurek® / Azathioprin |
| O | Synacthen® / Corticotropin |
| O | Sandimmun® / Cyclosporin |
| O | Endoxan® / Cyclophosphamid |
| O | Rituximab |
| O | Other |
|  | *---------------------------------------------* |

| **H8.** | **If you started immunomodulatory therapy based on the initial diagnosis consultation, how long did you take it?**  *Please select only one of the following answers.* |
| --- | --- |
|  |  |
| O | Less than 6 months |
| O | Between 6 months and a year |
| O | Between 1 and 2 years |
| O | Between 2 and 3 years |
| O | Stopped after more than 3 years |
| O | Initial therapy is still ongoing |

|  |  |
| --- | --- |

| 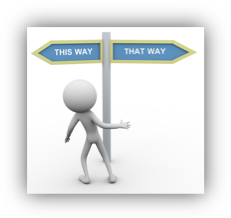 | *If you selected “Initial therapy is still ongoing” --> continue with question* ***H10****.*  *Otherwise --> continue with question* ***H9****.* |
| --- | --- |

| **H9.** | **If you stopped the initial treatment, what were the reasons for doing so?**  *Please select all applicable answers.* |
| --- | --- |
|  |  |
| O | Side effects |
| O | Planned pregnancy |
| O | Pregnancy occurred |
| O | Insufficient effect |
| O | Lack of confidence in the treatment |
| O | Other |
|  | If other, please specify |

|  |  |
| --- | --- |
